# Evidence for the Use of Patient-Reported Outcome Measures in the Treatment of Patients With Noncommunicable Diseases: Systematic Review

**DOI:** 10.2196/66160

**Published:** 2025-09-16

**Authors:** Marie Villumsen, Benedikte Irene von Osmanski, Kirsten Elisabeth Lomborg, Kirstine Skov Benthien

**Affiliations:** 1 Center for Clinical Research and Prevention Bispebjerg and Fredeiksberg Hospital University Hospital Copenhagen Frederiksberg Denmark; 2 Signum Life Science Copenhagen Denmark; 3 Steno Diabetes Center Copenhagen University Hospital Copenhagen Herlev Denmark; 4 Department of Clinical Medicine University of Copenhagen Copenhagen Denmark; 5 Department of Pulmonary Medicine and Endocrinology Hvidovre Hospital University Hospital Copenhagen Hvidovre Denmark

**Keywords:** patient-reported outcome measures, chronic obstructive pulmonary disease, diabetes, heart disease, inflammatory bowel disease, rheumatoid arthritis, noncommunicable diseases, non-communicable, health care decision-making, medical informatics, systematic reviews, review

## Abstract

**Background:**

The use of patient-reported outcome measures (PROMs) as a clinical tool for screening and decision-making has gained widespread interest, with numerous implementation activities across specialties, even though the evidence has not been clear until now.

**Objective:**

The aim of this study was to assess the evidence for using PROMs in clinical practice for patients with diabetes, chronic obstructive pulmonary disease (COPD), heart disease, rheumatoid arthritis (RA), and inflammatory bowel disease (IBD). Additionally, we sought to determine the characteristics of the most effective PROM interventions.

**Methods:**

We conducted a systematic review of published randomized controlled trials (RCTs) on the use of PROMs for clinical purposes, such as systematic PROM assessment alone or with a predefined PROM-based decision-making method. Eligible studies included adult patients (>18 years) with diabetes, COPD, heart disease, RA, or IBD. We excluded studies using PROMs as an outcome measure or otherwise not meeting the inclusion criteria. We searched the PubMed/MEDLINE, CINAHL, EMBASE, and Web of Science databases until February 2023. Two investigators independently screened titles, abstracts, and relevant full texts. Three investigators completed data extraction and risk-of-bias assessment using version 2 of the Cochrane risk-of-bias tool for randomized trials (RoB 2). The data were presented in a narrative synthesis and in summarized form.

**Results:**

The search yielded 21,203 papers, 686 (3.2%) full-text papers were screened, and 56 (8.2%) original studies were included in the review. The studies included patients with heart disease (n=17, 30.4%), COPD (n=13, 23.2%), diabetes (n=10, 17.9%), IBD (n=9, 16.1%), and RA (n=6, 10.7%), as well as patients with mixed diagnoses (n=1, 1.8%). All interventions incorporated systematic PROM assessments. Some interventions additionally used a predefined method for PROM-based decision-making (n=19, 33.9%) or PROM-based dialogue (n=9, 16.1%), while 5 (8.9%) interventions aimed to substitute face-to-face consultations. The predominant mode of PROM administration was over the phone, followed by electronic devices and apps. Endpoints included disease activity, health care use, mortality, mental well-being, quality of life, self-efficacy, self-care, daily functioning, and other outcomes. Six studies with a low risk of bias demonstrated a positive effect or noninferiority of the PROM intervention.

**Conclusions:**

The evidence base for clinical use of PROMs is sparse, with few studies evaluated to have a low or a medium risk of bias. The clinical use of PROMs does not appear superior to usual care in the five included chronic diseases on any endpoint. To guide further research, we highlighted 6 (10.7%) studies with a low risk of bias and PROM interventions with a positive effect. These were characterized by symptom assessment with predefined cutoffs used for decision and dialogue support.

**Trial Registration:**

PROSPERO CRD42021226896; https://www.crd.york.ac.uk/PROSPERO/view/CRD42021226896

## Introduction

The use of patient-reported outcome measures (PROMs) as endpoints in clinical trials is well established and recommended [1]. Furthermore, PROMs have gained interest as a tool to optimize patient-centered care and other supportive interventions that seek to include patients’ preferences and values [2-4]. PROMs are often self-completed questionnaires measuring symptoms, health-related quality of life (HRQoL), personal experience of health care, and health-related behaviors. In routine practice, it has been suggested that PROMs may increase health professionals’ awareness of and ability to address patients’ concerns [5]. Including PROMs in the electronic health record may trigger relevant clinical actions [6,7] and allow patients and health professionals to observe important trends over time and adjust the health care accordingly [8]. Therefore, questionnaires are continuously implemented in routine health care for systematic PROM assessments, informing clinical decisions and supporting the dialogue between patients and health care professionals (HCPs) [9,10].

Despite the promising aspects of using PROMs to improve health care, efforts to put theory into practice have met several barriers. When implementing PROMs in clinical practice, it is important to know the effectiveness, and this must be evaluated in relation to the time the patient spends completing the PROMs and the resources spent by HCPs. A recent systematic review found that patients may question the relevance and validity of PROMs, lack understanding of purpose, and find the clinical use inconsistent [11]. At the same time, HCPs may be concerned with adding undue burden, cause distress, or impact health care detrimentally if the correct infrastructure is not in place [12]. Furthermore, trials may fail to include the patients most burdened by symptoms [13].

The effectiveness of PROMs in cancer has been reviewed by Graupner et al [14], who demonstrated positive effects on several outcomes, including the HRQoL and survival, but also found included studies lacking power and with a high risk of bias. Although the evidence during acute cancer treatment has been described, the evidence in other noncommunicable diseases is unclear.

To close this gap, this systematic review aimed to address the following question: What is the evidence for using PROMs to improve health care, and what characterizes the most effective interventions? Specifically, the aim of this study was to (1) assess the evidence for using PROMs to improve any endpoint in diabetes, chronic obstructive pulmonary disease (COPD), heart failure (HF), ischemic heart disease, rheumatoid arthritis (RA), and inflammatory bowel disease (IBD) and (2) identify the characteristics of effective interventions that have been evaluated and documented in publications with a low or moderate risk of bias to propose directions for future research.

## Methods

### Study Design

We conducted this systematic review in accordance with a predefined protocol. The review was reported in line with the PRISMA (Preferred Reporting Items for Systematic Reviews and Meta-Analyses) statement (Multimedia Appendix 1) [15,16]. The review was registered at PROSPERO (International Prospective Register of Systematic Reviews; registration number CRD42021226896; date December 18, 2020).

### Eligibility Criteria

Only studies written in English were included. Studies selected for inclusion were found to meet the following PICOS (Population, Intervention, Comparison, Outcomes, and Study Design) framework (Table 1) [15]:

- Population: Eligible studies included adult patients (>18 years) with type 1 or type 2 diabetes, COPD, heart disease (HF or ischemic heart disease), RA, or IBD. These noncommunicable diseases were selected due to a high burden of disease measured by disability-adjusted life-years [17].
- Intervention: Eligible studies described any questionnaire used for assessing patient-reported health conditions in routine clinical practice, where the assessment results were forwarded to an HCP. Acceptable interventions could imply (1) systematic PROM assessment alone without a predefined plan for reacting to the responses, (2) systematic PROM assessment plus predefined PROM-based decision-making, (3) systematic PROM assessment as replacement of face-to-face visits, or (4) systematic PROM assessment implemented to support the dialogue between the HCP and the patient in a clinical health care setting.
- Comparison: Eligible studies described usual care alone or passive usage of PROMs (defined as PROM assessments where results were not forwarded to an HCP).
- Outcome measures: The studies were not restricted to specific outcomes.
- Study design: All randomized controlled trials (RCTs), including cluster RCTs and pilot studies, were included.

**Table 1:** Eligibility according to PICOS^a^ criteria for systematic selection of studies.

PICOS framework component	Inclusion criteria	Exclusion criteria
Population	Adult patients Diabetes COPD^b^ Heart disease RA^c^ IBD^d^	Pediatric patients Psychiatric patients Pregnant patients Surgical patients
Intervention	PROMs^e^ alone PROM-based decision-making PROM replacement of face-to-face visits PROM support of the dialogue	Intervention including wearables Multicomponent interventions
Comparison	Usual care alone Passive usage of PROMs	Active usage of PROMs Other measures
Outcomes	Not restricted to specific outcomes	—^f^
Study design	RCT^g^ Cluster RCT Pilot study	Observational study Literature review

^a^PICOS: Population, Intervention, Comparison, Outcomes, and Study Design.

^b^COPD: chronic obstructive pulmonary disease.

^c^RA: rheumatoid arthritis.

^d^IBD: inflammatory bowel disease.

^e^PROM: patient-reported outcome measure.

^f^Not applicable.

^g^RCT: randomized controlled trial.

### Search Strategy

The research team developed the search strategy. We sought advice from an information expert at the Royal Library to ensure all relevant Medical Subject Headings (MeSH) and filters were addressed. We conducted several pilot searches to capture targeted papers. The underlying principle of the search strategy is delineated in Textbox 1. The specific strategy and search strings are presented in Tables S1-S4 in Multimedia Appendix 2. We systematically searched the following electronic databases for potentially eligible papers: PubMed/MEDLINE, CINAHL, EMBASE, and Web of Science. The initial search was completed on December 18, 2020, for all databases and repeated on February 8, 2023, for PubMed/MEDLINE only (Table S5 in Multimedia Appendix 2).

**Principle for the search strategy.**

The search strategy was constructed around three primary components: disease, intervention, and study design.

- Disease: For the disease component, we used Medical Subject Headings (MeSH) terms and conducted searches within the titles and abstracts to identify pertinent diseases.
- Intervention: We specifically searched for patient-reported outcome measures (PROMs) by directly targeting patient-reported outcomes (PROs) and PROMs, along with their synonyms. Additionally, we included synonyms for quality of life, alongside measurement terms. Our preliminary searches revealed that PROMs are frequently administered via telemedicine; thus, we incorporated telemedicine in our search to ensure comprehensiveness.
- Study design: To identify randomized trials, we used Cochrane’s validated filter.

Titles and abstracts of the identified papers retrieved from electronic databases and other searches were exported to the systematic review management software Covidence, which was used solely to manage references and remove duplicates. No automated screening was performed in Covidence. Two reviewers independently screened all titles and abstracts according to the eligibility criteria. Full texts were reviewed if eligibility could not be determined by the title and abstract alone or in the case of disagreement. We made the final decision in agreement by going through all the included full texts in the group. The study selection process was reported in a flowchart.

### Risk of Bias in Individual Studies

Risk of bias was assessed by one of three of the authors independently using version 2 of the Cochrane risk-of-bias tool for randomized trials (RoB 2) [18]. The assessment was hereafter reviewed by one of the other authors. In the case of discrepancies, the risk-of-bias assessment was agreed on in a joint evaluation. One author also authored one of the included studies and was not involved in the risk-of-bias assessment.

### Data Synthesis

Data synthesis followed the synthesis without meta-analysis (SWiM) guidelines [19]. In short, the key characteristics of the studies were extracted and recorded in a predesigned table. If an intervention was reported in 2 or more papers, information about the first author and publication year from all papers were collapsed and reported under the primary publication. We used information from all papers to report the intervention and primary and secondary outcomes. Based on the extracted information, we created a narrative synthesis presenting the characteristics of the studies, the PROM characteristics, and the outcomes. The studies were grouped according to primary outcomes and study populations, and since the outcomes varied in content and type, data were synthesized with vote counting based on direction of effects. To ascertain the characteristics of the most effective PROM interventions, we categorized studies with a low or moderate risk of bias, reporting a positive effect if the intervention group demonstrated a statistically significant superior outcome compared to the control group or reporting noninferiority if the difference between the intervention and control groups was below the noninferiority threshold. The characteristics of these studies were synthesized separately. The PROSPERO protocol included assessment with GRADE (Grading of Recommendations Assessment, Development and Evaluation) [20]; however, this method was discarded because of intervention and outcome heterogeneity, so consistency of effects could not be assessed. More study details are described in Tables S6 and S7 in Multimedia Appendix 2.

## Results

### Study Details

**Figure 1:**
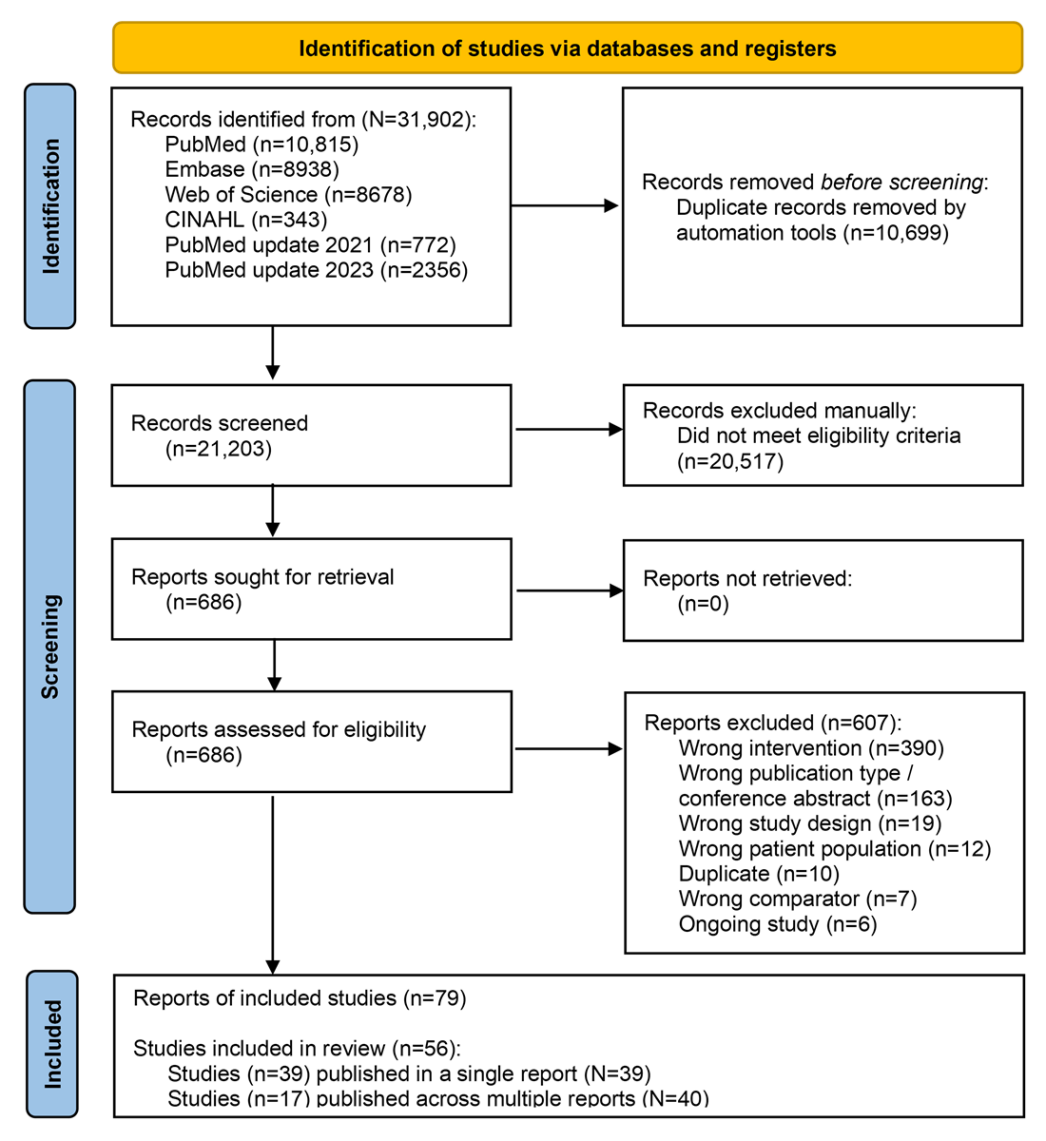
PRISMA flowchart for the study selection process.

The search identified 31,902 records. After identification of duplicates, 21,203 (66.5%) papers that were screened at the title/abstract level resulted in 686 (3.2%) potentially eligible papers. Following full-text assessment, 606 (88.3%) reports were excluded, mainly due to wrong interventions and publication types, and 56 trials described in 79 (13%) papers [21-99] were included (see the flowchart in Figure 1). The number of study participants varied widely (range: 17-1653 individuals, median 212.5 individuals). Of the 56 trials, 17 (30.4%) included patients with heart disease, 13 (23.2%) included patients with COPD, 10 (17.9%) included patients with diabetes, 9 (16.1%) included patients with IBD, 6 (10.7%) included patients with RA, and, finally, 1 (1.7%) included patients with mixed diagnoses (heart disease and diabetes).

### PROM Characteristics

Characteristics of the selected studies and PROMs are summarized in Table 2. Telephonic PROM administration was most common, followed by devices and applications (apps). The weighted mean age among participants receiving a PROM intervention delivered over the phone was 62.9 years (63 years for phone calls and 57.9 years for SMS). The first described use of devices for collection of PROMs was published in 2008. Apps for tablets and smartphones were introduced in 2007 and 2009, respectively. The participants’ weighted mean age was higher in interventions that used devices (71 years) than interventions using apps, webpages, or laptops (53.2 years).

**Table 2:** Overview of the characteristics of the included 56 trials reporting the results of RCTs^a^ of the use of PROMs^b^ in the clinic.

Study characteristics and categories		Studies (N=56), n (%)
**Disease**		
	COPD^c^	13 (23.2)
	Diabetes	10 (17.9)
	Heart disease	17 (30.4)
	Heart disease and diabetes	1 (1.8)
	IBD^d^	9 (16.1)
	RA^e^	6 (10.7)
**Continent**		
	Asia	1 (1.8)
	Europe	22 (39.3)
	North America	27 (48.2)
	Oceania	2 (3.6)
	South America	1 (1.8)
**Purpose**		
	Replace face-to-face visits	5 (8.9)
	Systematic PROM assessment alone	24 (42.9)
	Systematic PROM assessment and decision support	18 (32.1)
	Systematic PROM assessment and dialogue support	9 (16.1)
**Administration of PROM**		
	Device	10 (17.9)
	Interview	3 (5.4)
	Mobile app or tablet app	10 (17.9)
	Paper	3 (5.4)
	Telephone and SMS	16 (28.6)
	Unknown	3 (5.4)
	Web page or laptop	11 (19.6)
**PROM intervention validation**		
	No, unspecified questions	15 (26.8)
	No, specified questions	18 (32.1)
	Yes, validated scales	23 (41.1)
**Intervals between PROMs**		
	Daily or twice daily	20 (35.7)
	Weekly or biweekly	8 (14.3)
	Monthly	5 (8.9)
	Bimonthly to biyearly	5 (8.9)
	Before or at consultation	8 (14.3)
	Optional	4 (7.1)
	2-4 times	3 (5.4)
	1 time	3 (5.4)
**Feedback**		
	HCP^f^	8 (14.3)
	HCP by cutoff	25 (44.6)
	Patient and HCP	8 (14.3)
	Patient and HCP by cutoff	15 (26.8)

^a^RCT: randomized controlled trial.

^b^PROM: patient-reported outcome measure.

^a^COPD: chronic obstructive pulmonary disease.

^d^IBD: inflammatory bowel disease.

^e^RA: rheumatoid arthritis.

^f^HCP: health care professional.

All interventions included systematic PROM assessments, and some additionally included a predefined method for PROM-based decision-making (n=19, 33.9%) [21-39] or PROM-based dialogue (n=9, 16.1%) [40-48], and 5 (8.9%) interventions intended to replace face-to-face visits [49-53]. The PROM interventions used validated scales in 23 (41.1%) of the studies, while specified questions or unspecified questions were used in the remaining interventions. The content of the PROMs varied from symptoms and health status to the HRQoL, health beliefs, and self-care. All interventions included feedback to HCPs as per the inclusion criteria, while 23 (41.1%) also provided feedback to patients [22,27,28,30,32,33,36,37,39, 41-43,50,51,54-62]. In 39 (69.6%) of the interventions, predefined cutoff values were used to ensure feedback to the HCPs, 18 (46.2%) of which included alerts. Intervals between PROMs were not always documented but varied widely from a single assessment to collections twice daily. In the 20 (35.7%) interventions where PROMs were collected daily or twice daily, the number of questions varied from 1 to 15. Daily collection was used for COPD (n=9, 16.1%) [28,32,34,35,43,55,63-65], heart disease (n=9, 16.1%) [36,37,57-59,61,66,67], IBD (n=1, 1.8%) [23], and RA (n=1, 1.8%) [68] treatment. The daily PROM collection continued for 3-24 months. Studies only collecting PROMs once or with long intervals between had more comprehensive questionnaires.

Characteristics of the studies and interventions are presented in Tables S6 and S7 in Multimedia Appendix 2.

### Outcomes

All trials included more than one outcome. The following review of the results focuses on primary outcomes (Table S8 in Multimedia Appendix 2). Secondary outcomes are listed in Table S9 in Multimedia Appendix 2. Of the 56 trials, 12 (21.4%) had favorable results in the primary outcomes, 3 (5.4%) were noninferior, 6 (10.7%) had more than one primary outcome and mixed results, 6 (10.7%) were feasible, 1 (1.8%) was not feasible, 2 (3.6%) had no group comparison, 24 (42.9%) had no effect, and 2 (3.6%) had negative results by increasing hospital admissions with the intent of reducing them.

Of the 10 (17.9%) studies of patients with diabetes, 1 (10%) concluded noninferiority [53], 1 (10%) focused on feasibility [40], 1 (10%) demonstrated a mixed effect of PROMs [44], and the other 7 (70%) studies showed no effect [30,31,33,45, 47,69,99]. For studies of patients with COPD (n=13, 23.2%), there was a positive or partially positive effect in 6 (46.2%); in addition, 2 (66.7%) of 3 feasibility studies were feasible [64,65]. In the 18 (32.1%) studies including patients with heart disease, 5 (27.8%) were positive and 1 (5.6%) intervention was feasible [59]. There was a positive effect of the intervention in 33% of the studies for patients with IBD (n=9, 16.1%), 2 (22.2%) were feasible [23,39], and 1 (11.1%) was noninferior [52]. Half of the studies including patients with RA were positive, and 1 (16.7%) intervention was noninferior [50].

#### Disease Activity

In 13 (23.2%) studies, disease activity or remission was reported by biomarker thresholds [30,31,44,47,53,60], Boolean remission [92], disease activity indexes [21,22,34,50-52,56], or the time to reach the final dose of medical treatment [37].

None of 5 (8.9%) studies including patients with diabetes reported an effect on glycemic control [30,44,47,60]. However, in an intervention using PROMs to decide routine visits, the hemoglobin A_1c_ (HbA_1c_) levels were below the predefined noninferiority margin [53]. Biweekly automated telephone management was tested, with no effect on HbA_1c_ [30,31,60]. PROMs used to prioritize visits and facilitate discussion of psychological well-being [44] did not affect measures of HbA_1c_.

A study of patients with RA receiving PROM-based telehealth with graphical overview and automated decision support was noninferior to usual care [50]. An intervention with frequent PROM monitoring using SMS did not result in Boolean remission of patients with early RA [92].

Common for 5 (8.9%) studies on patients with IBD (n=9, 16.1%), the collected PROMs triggered alerts and action plans customized for intervention patients generated based on the responses. One showed a positive effect [21], another showed noninferiority [52], whereas the others did not show a difference between the intervention group and usual care [22,51,56].

A daily telehealth approach with PROM collection and feedback reduced disease progression in patients with COPD [34]. A similar intervention for patients with HF succeeded in a faster titration of carvedilol compared to usual care [37].

#### Health Care Use and Mortality

Hospital admission [61], readmission [49,67,72,96,97], time to admission [32,35,54], and outpatient visits [29,84] were outcomes in 11 (19.6%) studies. In addition, 1 (1.8%) study had mortality [89] as an outcome, and 6 (10.7%) studies used a composite of admission and mortality [24,25,36,55,76,91] as outcomes. Of 16 (28.6%) interventions evaluating the effects of PROMs on hospitalization, 3 (18.8%) reported a positive effect. A multicenter trial successfully reduced a composite endpoint of all-cause mortality and hospitalization for worsening HF in a telephone intervention using PROM to adjust treatment [25]. In contrast, no other PROM intervention studies reported effect on a composite outcome of hospital admission and death in patients with HF [24,36,76,91] or COPD [55].

An effective intervention for reducing the mean number of outpatient visits for patients with IBD included monthly reporting of PROMs into an app for tablets or smartphones with alerts prompting outpatient visits. The system intensified monitoring modules in the case of flares [84].

Two interventions used daily PROMs to generate alerts to prevent admission due to COPD exacerbations [32,35]. No difference was seen when comparing the intervention with usual care [32], whereas the time to hospitalization was shorter when comparing active feedback with passive PROMs [35]. For patients with HF, an underpowered study of daily PROMs indicated a lower mean time to the first HF-related hospitalization [54].

Two interventions using PROMs for systematic PROM assessment alone without a predefined plan for reacting to the responses did not find a reduction in hospitalization [96,97]. An intervention with daily PROMs collected via voice-activated technology had an unexpected increase in the number of hospitalizations and emergency department visits in participants with HF [61]. Two studies resulted in a higher number of readmissions using daily PROMs in patients with COPD [49] and patients with diabetes and HF [72]. A study on RA found that monitoring PROMs could reduce the number of physical visits, while maintaining tight control of disease activity [29].

#### Mental Well-Being

In patients with diabetes, PROMs have been used to screen for diabetes distress [40], psychological well-being [44], and depression [33,45]. In 2 (50%) studies, participants completed electronic PROMs prior to consultation, which did not show an effect on diabetes distress [40] or the depression score [33]. A favorable effect on mood was reported from an intervention using PROMs on psychological well-being as part of routine outpatient care [44]. In an intervention about self-care behavior and tailored talking points about emotional health, both control and intervention groups continued to have moderate-to-severe depression symptoms [45].

#### Health-Related Quality of Life

In studies aiming to improve the HRQoL using a PROM intervention (n=10, 17.7%), 2 (20%) studies showed an improvement [41,46], 1 (10%) showed noninferiority [52], 1 (10%) had mixed effects [63], 4 (40%) showed no effect [22,26,56,69], and 2 (20%) showed an unfavorable effect [49,71] compared to usual care. A multicenter trial found a significantly better health status favoring patients with COPD reporting PROMs of general well-being, symptoms, and medications. Alerts were reviewed by HCPs twice weekly, and patients were contacted either via messages or over the phone if any action was needed [63]. Automated telephone-based symptom and side effect monitoring had no effect on the quality of life (QoL) in patients with diabetic peripheral neuropathy [69].

Weekly monitoring of symptoms and medications in patients with IBD did not improve disease-specific QoL [22,56]. However, noninferiority was reported in a similar intervention with quarterly and as-needed PROM monitoring [52]. No effect was documented on quality-adjusted life-years for patients with acute coronary syndrome without a history of depression receiving systematic depression symptoms assessment with or without providing depression treatment compared to usual care [26].

#### Self-Efficacy, Self-Care, and Daily Functioning

Of 5 (8.9%) studies evaluating the use of PROMs to increase daily functioning [41,99], self-care [43], or self-efficacy [27,57], 4 (80%) showed positive results. In patients with HF, daily PROM collection over the phone increased self-efficacy [57]. Self-efficacy also improved among patients with RA when using PROMs prior to usual consultations with a nurse who provided patient education [27]. Self-care behavior improved in patients with COPD who answered questions on symptoms at least four times a week via a smartphone app that alerted HCPs [43]. Receiving personalized feedback on responding to somatic and psychosocial PROMs improved social participation in patients with IBD [41]. A cluster randomized trial concluded that using PROMs was inconsistent with the nurse-led detection of distress and daily functioning in routine practice [99].

#### Other Outcomes

Other outcomes included feasibility [23,49,59,64,65], patient satisfaction [42,62], quality of care [44,48,84], physician-patient interaction [42], and health care costs [21,28]. PROM interventions were considered feasible in 5 (8.9%) studies [23,39,59,64,65], and 1 (1.8%) intervention was considered unfeasible [49]. In 2 (22.2%) interventions for patients with IBD, the intervention was appreciated by compliers, but adherence was low [23,39], and 2 (33.3%) studies of patients with RA found that using PROMs before health care visits has no impact on patient satisfaction [42,62]. Monitoring of phycological well-being in outpatients with diabetes did not change the overall evaluation of the quality of diabetes care [44]. PROMs of psychosocial needs and priorities before routine consultations with a COPD nurse allowed shared decisions about self-management support and increased the quality of care in patients with COPD [48]. Collecting PROMs daily from patients with HF was deemed feasible by the authors, even though number of readmissions was similar in the two groups [59]. A monthly PROM-based self-management system did not enhance patient-reported quality of care [84].

Two studies evaluated cost savings [21,28]. Using daily PROMs of symptoms and medications in the treatment of COPD led to significant cost savings in a telephone intervention [28]. An intervention with proactive symptom monitoring was without a significant difference in median annual IBD-related health care charges [21].

### Adverse Events

Several studies indicated adverse events [23,38,39,49,61, 65,71,72,89]. These included 2 (22.2%) studies in patients with heart disease and diabetes, which intended to reduce hospital admissions but increased the number instead [61, 72], and 1 (11.1%) study in which the subgroup analyses indicated increased mortality in women [89]. Adverse events were also observed in secondary outcomes: decreased peak performance and physical activity [65], increased hospital admissions [38], increased medical treatment [39], deterioration in symptoms [71], increased symptoms of depression [23], and higher costs [49].

### Characteristics of Effective PROMs

The predefined secondary aim of this systematic review was to describe effective PROM interventions with acceptable quality defined by a low risk of bias or some concerns. Excluding a study [24] that was later debunked by a larger study [89], 6 (10.7%) studies had positive results in the primary endpoint, had positive results in one of two primary endpoints, or were noninferior in replacing face-to-face sessions [25,42,46,50,53,57]. The PROM interventions used multiple administration forms and were focused on symptoms of disease, and 5 (83.3%) of the 6 studies included support for decisions and dialogue [25,42,46,50,53], 4 (66.7%) studies included validated PROMs [42,46,50,53], and 2 (33.3%) included specified questions [25,57]. In addition, 5 (83.3%) of the 6 had 2-week to 6-month intervals between PROM collection [25,42,46,50,53], and 4 (66.7%) of the 6 studies included feedback to patients as well as HCPs, with 3 (75%) having a graphical display of the results [42,46,50]. Furthermore, 2 (33.3%) studies included patients with heart disease [25,57], 2 (33.3%) included patients with RA [42,50], 1 (16.7%) included patients with diabetes [53], and 1 (16.7%) included patients with COPD [46]. Positive effects were achieved in the HRQoL [46], physician-reported interaction [42], self-efficacy [57], a composite endpoint with hospital admissions and death [25], and noninferior RA or diabetes activity when replacing outpatient visits with PROMs [50,53]. These 6 (10.7%) studies had little loss to follow-up, and 5 (83.3%) of the 6 studies had more study consenters than study decliners [25,42,46,50,53].

### Risk of Bias

**Figure 2:**
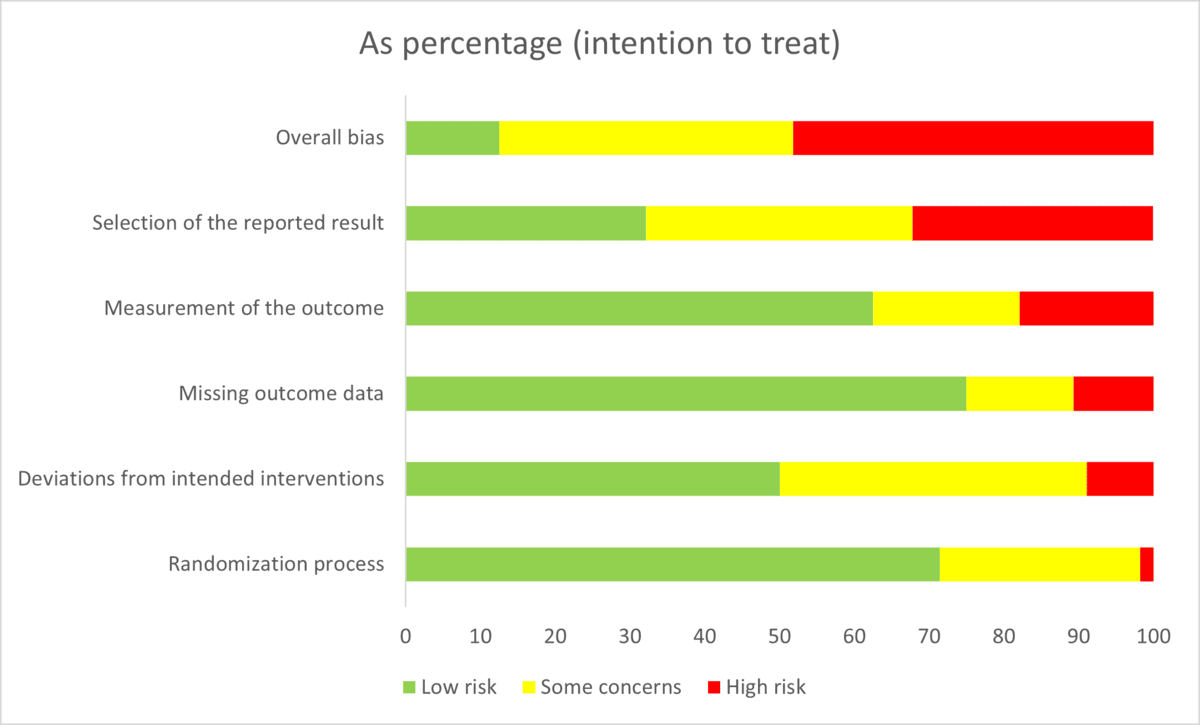
Risk of bias across all included studies using RoB 2. RoB 2: version 2 of the Cochrane risk-of-bias tool for randomized trials.

Seven studies were rated with an overall low risk of bias (Figure 2). Among the 22 (39.3%) studies with some concerns regarding risk of bias, concerns relating to the reporting of results were the most frequent (n=13, 59.1%), followed by concerns arising from deviations from the intended intervention (n=10, 45.5%), the randomization process (n=9, 40.9%), the measurement of the outcome (n=5, 22.7%), or missing outcome data (n=5, 22.7%). Nearly half of the studies had a high risk of bias (n=27, 48.2%). No studies had biases in all domains, but 2 (3.6%) studies had high risk in four of five domains [27,38]. The most frequent domain for a high risk of bias was selection of reported results (n=18, 32.1%) led by a lack of prospective descriptions of the trials in registries or protocol papers. Among studies with a low risk of bias or some concerns, 14.3% (4/28) had positive results, while among studies with a high risk of bias, 33.3% (9/27) had positive results. Risk-of-bias evaluation of the individual studies can be found in Table S8 in Multimedia Appendix 2.

## Discussion

### Principal Findings

This systematic literature review identified sparse evidence regarding the effectiveness of the clinical use of PROMs in patients with five noncommunicable diseases: diabetes, COPD, heart disease, RA, and IBD. Considering the increase in PROMs’ popularity and the fact that we included five highly prevalent and burdensome diseases [100-102], it is notable that the literature search only revealed 56 trials in total, half of which had a high risk of bias. The included RCTs investigated a wide range of outcomes, thus producing a heterogeneous set of data and indicating a lack of consensus on the role of PROMs in clinical practice. Of the 56 RCTs, 12 presented positive results in the primary endpoint, 6 had mixed results in multiple primary endpoints, and 3 were noninferior. Focusing on the 28 RCTs with a low risk of bias or some concerns, 4 resulted in a better primary outcome and 2 demonstrated noninferiority. Successful interventions were characterized by using validated or specific PROMs and had little loss to follow up. They provided feedback to patients as well as HCPs, with 3 interventions incorporating a graphical display of the results.

### Comparison With Prior Work

Compared to the results of this review, the results of reviews about acute cancer treatment are summarized as positive [14,103]. However, the majority of the included studies lacked power to detect group differences, and the conclusion of predominantly positive findings may be questioned [14]. The conditions of acute cancer treatment may also differ from those of long-term diseases where patients may have had more time to recognize, to learn self-management of symptoms and side effects, and to seek relevant health care.

### Methodological Considerations

This review was conducted with screening by two independent researchers, and discrepancies were handled by including a third researcher in a consensus approach to ensure internal validity. The limited descriptions of interventions posed certain constraints, often lacking detail, which may have affected the external validity of this review. Adopting a more conservative approach of including only pure PROM interventions with no objective measurements at all would have reduced the number of eligible papers and the clinical relevance. Conversely, a less stringent approach, including more interventions where PROMs were one of multiple components, would have reduced focus and confidence in the results. Second, the heterogeneity of the studied PROM interventions and outcomes prevented meta-analyses and permitted only vote counting for data synthesis, which does not allow for differential weights to be allocated to each study.

The proportion of papers included in the review to the original number of studies identified in the search was less than 0.3%. The likely cause was the intervention search words that did not allow for distinction between RCTs using PROMs as endpoints and RCTs using PROMs for intervention. Attempts to reduce the number of papers for screening would have excluded relevant papers, which is why the searches were placed at a high level of sensitivity. In comparison, the systematic review by Graupner et al [14] about PROMs focusing on cancer revealed a quite similar proportion of included studies as 22 of 8341 identified studies were included.

### Balancing Person-Centered Care With Evidence-Based Practice

The expectations toward PROMs are underlined by the implementation activities that have preceded robust evidence. Implementation before evaluation demonstrates the dissimilar approach toward PROM interventions to that of drug interventions that would have required robust trials before being released into the market. As demonstrated in this review, adverse effects may also occur in PROM interventions. Furthermore, PROMs require time and resources of HCPs as well as patients, and robust evaluations of PROMs are urgently needed.

PROMs are expected to support a person-centered approach. With 3 noninferiority trials, most trials evaluated superiority in a wide range of endpoints. The range of endpoints underscore the unclear expectations toward PROMs and the difficulty in operationalizing patient-centeredness. Noninferiority trials may be preferable if the purpose of PROMs is to replace face-to-face outpatient visits to compensate for the resources spent on PROM administration and implementation. However, trials to demonstrate noninferiority with a clinically acceptable margin may not be feasible to conduct [104]. Furthermore, most PROM interventions are designed as an addition to usual care, in which case only demonstrated superiority would merit spending resources on PROMs.

### Future Directions

The 6 effective PROM interventions may serve as inspiration for further development. They focused on symptoms and were mostly used for systematic PROM assessment, plus a predefined PROM-based decision support method or dialogue support as opposed to systematic PROM assessment alone. This underscores the significance of the organizational structure to ensure consistent monitoring of PROMs and suitable predefined clinical actions. The effective PROM interventions had little loss to follow up and few study decliners, which is a testament to the acceptability of the interventions. A review of patients’ reasons for not using digital PROMs has demonstrated the significance of patients’ health (patients would forego PROMs if they were too well or too sick) and that it could be burdensome to be confronted with one’s poor health and that technical problems, a lack of skills, language problems, and uncertainty about data security could all act as barriers toward the use of digital PROMs [105]. Patients with a moderate symptom burden who use PROMs could be referred to health care services at the expense of the most burdened patients who opt out of using PROMs, thereby increasing health care inequality.

Adding PROMS to usual care does not appear superior; however, replacing specific face-to-face visits with PROMS seems noninferior in patients with diabetes [53], IBD [52], and RA [50]. Noninferiority trials may be more suitable when PROMs are intended to replace face-to-face outpatient visits, thereby offsetting the resources allocated to the administration and implementation of PROMs.

### Conclusion

The evidence supporting the clinical use of PROMs in the five chronic diseases included in this study is limited. In these diseases, the clinical application of PROMs does not seem to offer any advantage over standard care in terms of any endpoint. To derive insights from successful research, we highlighted 6 PROM interventions that demonstrated positive effects and exhibited a low risk of bias. These were characterized by symptom assessment with predefined cutoffs used for decision and dialogue support.

## Appendix

Multimedia Appendix 1 PRISMA checklist.

Multimedia Appendix 2 Search strategy, data synthesis, and additional results.

## Abbreviations

COPD: chronic obstructive pulmonary disease
HbA_1c_: hemoglobin A_1c_
HCP: health care professional
HF: heart failure
HRQoL: health-related quality of life
IBD: inflammatory bowel disease
PICOS: Population, Intervention, Comparison, Outcomes, and Study Design
PRISMA: Preferred Reporting Items for Systematic Reviews and Meta-Analyses
PROM: patient-reported outcomes measure
PROSPERO: International Prospective Register of Systematic Reviews
QoL: quality of life
RA: rheumatoid arthritis
RCT: randomized controlled trial
RoB 2: version 2 of the Cochrane risk-of-bias tool for randomized trials

## Data Availability

All data generated and analyzed during this study are included in this published paper and in Multimedia Appendix 2. The databases created during the data extraction process are available from the corresponding author upon reasonable request.
