# Supplementary material for: Evidence for the Use of Patient-Reported Outcome Measures in the Treatment of Patients With Noncommunicable Diseases: Systematic Review
Source: JMIR Med Inform. 2025 Sep 16;13:e66160. doi: 10.2196/66160 (PMC12485264; doi:10.2196/66160)
Supplement: Multimedia Appendix 2 [file medinform_v13i1e66160_app2.pdf]

# SUPPLEMENTARY

## EVIDENCE FOR THE USE OF PATIENT-REPORTED OUTCOME MEASURES IN THE TREATMENT OF PATIENTS WITH NON-COMMUNICABLE DISEASES: A SYSTEMATIC REVIEW

# TABLE OF CONTENTS

Table of Contents ........ 2
Table of tables ........ 2
Inclusion criteria ........ 3
Population ........ 3
Intervention ........ 3
Comparator ........ 3
Outcome measures ........ 3
Study design ........ 3
Search Strategy ........ 3
Search Results ........ 9
Data synthesis ........ 9
Grouping of the studies ........ 9
Standardized metrics and transformation ........ 9
Prioritize results for summary and synthesis ........ 9
Heterogeneity in reported effects ........ 10
Certainty of evidence ........ 10
Data presentation methods ........ 10
Results ........ 11
References ........ 43

# TABLE OF TABLES.

Table S1. Keywords used to develop the search strategy for PubMed. ........ 4
Table S2. CINAHL search strings. ........ 6
Table S3. EMBASE search strings. ........ 7
Table S4. Web of Science search strings. ........ 8
Table S5. Overview of literature searches. ........ 9
Table S6. Characteristics of included studies ........ 11
Table S7. Characteristics of the interventions in the included studies. ........ 19
Table S8. Risk of bias and primary outcomes of included studies. ........ 28
Table S9. Secondary outcomes of included studies. ........ 34

# INCLUSION CRITERIA

Only studies written in English were included. Studies selected for inclusion were found to meet the following PICOS [Population, Intervention, Comparison, Outcomes, and Study design] criteria [15]:

## POPULATION

Eligible studies included adult patients (>18 years) with diabetes, chronic obstructive pulmonary disease (COPD), heart failure (HF), ischemic heart disease, rheumatoid arthritis (RA), or inflammatory bowel disease (IBD). Studies solely involving pediatric patients, patients with psychiatric conditions, pregnant patients, or patients undergoing surgery were excluded.

## INTERVENTION

A PROM was defined as the use of a questionnaire of a patient-reported health condition in routine clinical practice for systematic PROM assessment alone, PROM assessment as replacement of face-to-face visits, a predefined PROM-based decision-making method, or systematic PROM-based dialogue support method between the healthcare professional and the patient.

Eligible studies described any PROM used in a clinical healthcare setting. Since PROMs should reflect the health condition as perceived by the patient, this includes questionnaires to assess symptoms, adverse drug reactions, level of function, physical or mental state, or HRQoL. Responses should feed back to healthcare professionals and be applied in routine clinical practice to assess patients for symptoms, optimize medical treatment and adverse effects, support patient-clinician communication, or decide on treatment plans. The clinical use of PROMs should be the main active intervention component for a study to be eligible. Multi-component interventions where clinical use of PROMs was one among several active intervention components (e.g., patient education, medication, or rehabilitation) were excluded. Furthermore, studies focusing on wearables or monitoring of vital signs, health behavior, or other objective measures that can be observed without including patient perception were excluded.

## COMPARATOR

Controls in eligible studies either received usual care alone or used PROMs as the intervention group but in a clinically passive way where information obtained in the PROMs was not passed forward to the clinicians and not used in the clinical care.

## OUTCOME MEASURES

Inclusion was not restricted to specific outcomes. Outcome measures in included studies could include patient-reported outcomes (e.g., HRQoL, satisfaction, or symptom burden), healthcare contacts (e.g., hospital admissions or outpatient contacts), patient-clinician communication, mortality, quality of care, and other outcomes.

## STUDY DESIGN

All randomized controlled trials (RCTs) were eligible, including cluster RCTs and pilot studies.

# SEARCH STRATEGY

The search strategy was developed to be suited for searched in Databases were searched using keywords and combinations of predefined keywords. Keywords were grouped by category (patients, intervention, and study design) using Boolean operators (AND/OR/NOT) and combined across categories to increase the

specificity of the search. We did not search specifically on the inclusion criteria comparator and outcome. Used keywords are presented in Table S1 and below the entire search string for the PubMed search in Textbox S1. Based on this search string, we tailored search strings to each of the other databases CINAHL (Table S2), EMBASE (Table S3), and Web of Science (Table S4).

**Table S1.** KEYWORDS USED TO DEVELOP THE SEARCH STRATEGY FOR PUBMED.

| **Patient/Population** | **Intervention** | **Study design** |
| --- | --- | --- |
| <u>Diabetes</u><br>• Diabetes Mellitus [MeSH]<br>• Diabetes [TiAb]<br>• NOT insipidus [TiAb]<br>• NOT gestational [TiAb]<br><u>Heart failure</u><br>• Heart failure [MeSH]<br>• Heart failure [TiAb]<br>• Cardiac failure [TiAb]<br>• Cardiac insufficiency [TiAb]<br>• Heart insufficiency [TiAb]<br><u>Ischemic heart disease</u><br>• Myocardial ischemia [MeSH]<br>• Myocardial ischemia* [TiAb]<br>• Myocardial ischaemia* [TiAb]<br>• Ischemic heart disease [TiAb]<br>• Ischaemic heart disease [TiAb]<br>• Coronary artery disease [TiAb]<br>• Myocardial infarction [TiAb]<br><u>Chronic Obstructive pulmonary disease</u><br>• Pulmonary Disease, Chronic Obstructive [MeSH]<br>• Chronic obstructive lung disease [TiAb]<br>• Chronic obstructive pulmonary disease [TiAb]<br>• COPD [TiAb]<br><u>Rheumatoid arthritis</u><br>• Arthritis, Rheumatoid [MeSH]<br>• Rheumatoid arthritis [TiAb]<br><u>Inflammatory Bowel Disease</u><br>• Inflammatory bowel diseases [MeSH]<br>• Inflammatory bowel disease* [TiAb]<br>• IBD [TiAb]<br>• Ulcerative colitis [TiAb]<br>• Crohns disease [TiAb]<br>• Crohn's disease [TiAb]<br>• Crohn disease [TiAb] | Patient reported outcome measures [MeSH]<br><br>Self report [MeSH]<br><br>Patient report* [TiAb]<br><br>Patient assess*[TiAb]<br><br>Self assess*[TiAb]<br><br>Self report* [TiAb]<br><br>(Quality of life [TiAb] OR Well-being [TiAb] OR Distress [TiAb] OR symptom* [TiAb]) **AND:**<br>• Assessment* [TiAb] OR<br>• Diary [TiAb] OR<br>• Diaries [TiAb] OR<br>• Questionnaire* [TiAb] OR<br>• Inventory [TiAb] OR<br>• Inventories [TiAb] OR<br>• Screen*[TiAb] OR<br>• Monitor* [TiAb]<br><br>Telemedicine [MeSH]<br><br>Telemedicine [TiAb]<br><br>Telemonitoring [TiAb] | **Cochrane highly sensitive search strategies for identifying randomized trials in PubMed; sensitivity- and precision-maximizing version (2008 revision)**<br><br>Link: https://work.cochrane.org/pubmed<br><br>**#1** randomized controlled trial [pt]<br>**#2** controlled clinical trial [pt]<br>**#3** randomized [tiab] OR randomised [tiab]<br>**#4** placebo [tiab]<br>**#5** clinical trials as topic [mesh: noexp]<br>**#6** randomly [tiab]<br>**#7** trial [ti]<br>**#8** #1 OR #2 OR #3 OR #4 OR #5 OR #6 OR #7<br>**#9** animals [mh] NOT humans [mh]<br>**#10** #8 NOT #9<br><br>We have added "Randomised" in line #3 to the original Cochrane version. |

### TEXTBOX S1. PUBMED SEARCH STRING.

((((diabetes mellitus[MeSH Terms] OR Diabetes[Title/Abstract]) NOT (Insipidus[Title/Abstract] OR Gestational[Title/Abstract])) OR (myocardial ischemia[MeSH Terms] OR myocardial ischemia*[Title/Abstract] OR myocardial ischaemia*[Title/Abstract] OR ischemic heart disease[Title/Abstract] OR ischaemic heart disease[Title/Abstract] OR coronary artery disease[Title/Abstract] OR myocardial infarction[Title/Abstract] OR heart failure[MeSH Terms] OR heart failure[Title/Abstract] OR cardiac failure[Title/Abstract] OR cardiac insufficiency[Title/Abstract] OR heart insufficiency[Title/Abstract]) OR (pulmonary disease, chronic obstructive[MeSH Terms] OR chronic obstructive pulmonary disease[Title/Abstract] OR chronic obstructive lung disease[Title/Abstract] OR COPD[Title/Abstract]) OR (arthritis, rheumatoid[MeSH Terms] OR rheumatoid arthritis[Title/Abstract]) OR (inflammatory bowel diseases[MeSH Terms] OR inflammatory bowel disease*[Title/Abstract] OR IBD[Title/Abstract] OR ulcerative colitis[Title/Abstract] OR crohns disease[Title/Abstract] OR crohn disease[Title/Abstract] OR crohn s disease[Title/Abstract])) AND (patient reported outcome measures[MeSH Terms] OR self report[MeSH Terms] OR patient report*[Title/Abstract] OR patient assess*[Title/Abstract] OR self assess*[Title/Abstract] OR self report*[Title/Abstract] OR ((quality of life[Title/Abstract] OR well-being[Title/Abstract] OR distress[Title/Abstract] OR symptom*[Title/Abstract]) AND (assessment*[Title/Abstract] OR Diary[Title/Abstract] OR Diaries[Title/Abstract] OR questionnaire*[Title/Abstract] OR Inventory[Title/Abstract] OR Inventories[Title/Abstract] OR screen*[Title/Abstract] OR monitor*[Title/Abstract])) OR (Telemedicine[MeSH Terms] OR Telemedicine[Title/Abstract] OR Telemonitoring[Title/Abstract])) AND ((randomized controlled trial[Publication Type] OR controlled clinical trial[Publication Type] OR randomized[Title/Abstract] OR randomised[Title/Abstract] OR placebo[Title/Abstract] OR clinical trials as topic[MeSH Terms:noexp] OR randomly[Title/Abstract] OR trial[Title]) NOT (animals[MeSH Terms] NOT humans[MeSH Terms]))) AND (english[Filter])

Table S2. CINAHL SEARCH STRINGS.

| **Chronic diseases** | **Patient reported outcomes** | **Controlled Trials** |
|---|---|---|
| MH Diabetes Mellitus OR AB diabetes NOT AB (insipidus OR gestational) | S1MH Patient-reported outcomes OR AB patient report* OR AB Self report* OR AB Patient assess* OR AB Self assess* OR AB Self report*<br>S2MH Quality of Life OR AB Quality of life OR AB Well-being OR AB Distress OR AB symptom*<br>S3AB Assessment* OR AB Diary OR AB Diaries OR AB Questionnaire* OR AB Inventory OR AB Inventories OR AB Screen* OR AB Monitor*<br>S4MH Telehealth OR AB Telehealth OR AB Telemedicine OR AB Telemonitoring<br>S5 S2 AND S3<br>S6 S1 OR S3 OR S4 OR S5 | S1MH randomized controlled trials<br>S2MH double-blind studies<br>S3MH single-blind studies<br>S4MH random assignment<br>S5MH pretest-posttest design<br>S6MH cluster sample<br>S7TI (randomised OR randomized)<br>S8AB (random*)<br>S9TI (trial)<br>S10MH (sample size) AND AB (assigned OR allocated OR control)<br>S11MH (placebos)<br>S12PT (randomized controlled trial)<br>S13AB (CONTROL W5 GROUP)<br>S14MH (CROSSOVER DESIGN) OR MH (COMPARATIVE STUDIES)<br>S15AB (CLUSTER W3 RCT)<br>S16MH ANIMALS+<br>S17MH (ANIMAL STUDIES)<br>S18TI (ANIMAL MODEL*)<br>S19S16 OR S17 OR S18<br>S20MH (HUMAN)<br>S21S19 NOT S20<br>S22S1 OR S2 OR S3 OR S4 OR S5 OR S6 OR S7 OR S8 OR S9 OR S10 OR S11 OR S12 OR S13 OR S14 OR S15<br>S23 S22 NOT S21 |
| MH Heart Failure OR AB Heart failure OR AB cardiac failure OR AB cardiac insufficiency OR AB heart insufficiency<br>OR<br>MH Myocardial Ischemia OR AB Myocardial ischemia OR AB Ischemic heart disease OR AB Coronary artery disease OR AB Myocardial infarction | | |
| MH Pulmonary Disease, Chronic Obstructive OR AB Chronic obstructive lung disease OR AB Chronic obstructive pulmonary disease OR AB COPD | | |
| MH Arthritis, Rheumatoid OR AB Rheumatoid arthritis | | |
| MH Inflammatory bowel diseases OR AB Inflammatory bowel disease* OR AB IBD OR AB Ulcerative colitis OR AB Crohn* disease | | |

**Table S3. EMBASE SEARCH STRINGS.**

<table>
<tr><th>Chronic diseases</th><th>Patient reported outcomes</th><th>Controlled Trials</th></tr>
<tr><td>exp diabetes mellitus/<br><br>(Diabetes not insipidus).ti,ab,kw.</td><td rowspan="6">exp patient-reported outcome/<br><br>exp self report/<br><br>exp self monitoring/<br><br>(Patient report* or Patient assess* or Self report* or Self assess*).ti,ab,kw.<br><br>((Quality of life or Well-being or distress or symptom*) adj4 (Assessment* or Diary or Diaries or Questionnaire* or Inventory or Inventories or Screen* or Monitor*)).ti,ab,kw.<br><br>exp telehealth/<br>(telehealth or telemedicine or telemonitoring).ti,ab,kw.</td><td rowspan="6">1. (random$ or placebo$ or single blind$ or double blind$ or triple blind$).ti,ab.<br>2. RETRACTED ARTICLE/<br>3. or/1-2<br>4. (animal$ not human$).sh,hw.<br>5. (book or conference paper or editorial or letter or review).pt. not exp randomized controlled trial/<br>6. (random sampl$ or random digit$ or random effect$ or random survey or random regression).ti,ab. not exp randomized controlled trial/<br>7. 3 not (4 or 5 or 6)</td></tr>
<tr><td>exp rheumatoid arthritis/<br><br>(rheumatoid adj3 arthritis).ti,ab,kw.</td></tr>
<tr><td>exp heart failure/<br><br>(Heart failure or Cardiac failure or Cardiac insufficiency or Heart insufficiency).ti,ab,kw.<br><br>exp ischemic heart disease/<br><br>(Myocardial ischemia* or Ischemic heart disease or Coronary artery disease or Myocardial infarction).ti,ab,kw.</td></tr>
<tr><td>exp chronic obstructive lung disease/<br><br>(Chronic obstructive lung disease or Chronic obstructive pulmonary disease or COPD).ti,ab,kw.</td></tr>
<tr><td>exp inflammatory bowel disease/<br><br>Inflammatory bowel disease.ti,ab,kw.<br><br>IBD.ti,ab,kw.<br><br>Ulcerative colitis.ti,ab,kw.<br><br>(Crohns disease or Crohn's disease or Crohn disease).ti,ab,kw.</td></tr>
</table>

**Table S4. WEB OF SCIENCE SEARCH STRINGS.**

| Search | Search string |
|---|---|
| **#1** | TS=Diabetes |
| **#2** | TI=("Heart failure" OR "Cardiac Failure" OR "Heart insufficiency" OR "Cardiac insufficiency") OR<br>AB=("Heart failure" OR "Cardiac Failure" OR "Heart insufficiency" OR "Cardiac insufficiency") |
| **#3** | TS=("Heart failure" OR "Cardiac Failure" OR "Heart insufficiency" OR "Cardiac insufficiency") |
| **#4** | TI=("Myocardial ischemia" OR "Ischemic heart" OR "Coronary artery" OR "Myocardial infarction") OR<br>AB=("Myocardial ischemia" OR "Ischemic heart" OR "Coronary artery" OR "Myocardial infarction") |
| **#5** | TS=("Myocardial ischemia" OR "Ischemic heart" OR "Coronary artery" OR "Myocardial infarction") |
| **#6** | TI=("chronic obstructive lung" OR "chronic obstructive emphysema" OR "chronic obstructive pulmonary" OR "COPD") OR<br>AB=("chronic obstructive lung" OR "chronic obstructive emphysema" OR "chronic obstructive pulmonary" OR "COPD") |
| **#7** | TS=("chronic obstructive lung" OR "chronic obstructive emphysema" OR "chronic obstructive pulmonary" OR "COPD") |
| **#8** | TI=(Rheumatoid and arthritis) OR AB=(Rheumatoid and arthritis) |
| **#9** | TS=(Rheumatoid and arthritis) |
| **#10** | TI=("Inflammatory bowel disease") OR AB=("Inflammatory bowel disease") |
| **#11** | TS=("Inflammatory bowel disease") |
| **#12** | TI=("Crohns disease" OR "Crohn's disease" OR "Crohn disease" OR "Morbus Crohn") OR AB=("Crohns disease" OR "Crohn's disease" OR "Crohn disease" OR "Morbus Crohn") |
| **#13** | TS=("Crohns disease" OR "Crohn's disease" OR "Crohn disease" OR "Morbus Crohn") |
| **#14** | TI=("Ulcerative colitis") OR<br>AB=("Ulcerative colitis") |
| **#15** | TS=("Ulcerative colitis") |
| **#16** | TI=("Patient reported outcome" OR "Self report" OR "Patient report*" OR "Patient assess*" OR "Self assess*" OR "Self report*") OR<br>AB=("Patient reported outcome" OR "Self report" OR "Patient report*" OR "Patient assess*" OR "Self assess*" OR "Self report*") |
| **#17** | TS=("Patient reported outcome" OR "Self report" OR "Patient report*" OR "Patient assess*" OR "Self assess*" OR "Self report*") |
| **#18** | AB=(("Quality of life" OR Well-being OR Distress OR symptom* ) AND (Assessment* OR Diary OR Diaries OR Questionnaire* OR Inventory OR Inventories OR Screen* OR Monitor*)) |
| **#19** | TS=("Clinical trial" OR randomised OR randomized OR randomisation OR randomisation OR<br>placebo* OR (random* AND (allocat* OR assign*)) OR (blind* AND<br>(single OR double OR treble OR triple))) |
| **TI+AB** | (#1 OR #2 OR #4 OR #6 OR #8 OR #10 OR #12 OR #14) AND (#16 OR #18) AND #19 |
| **TS** | (#1 OR #3 OR #5 OR #7 OR #9 OR #11 OR #13 OR #15) AND (#17 OR #18) AND #19 |

# SEARCH RESULTS

The initial search of PubMed, CINAHL, EMBASE, and Web of Science was completed on December 18th, 2020. Because all papers included in the review from the initial search were identified in the PubMed database, the repeated searches on September 10th, 2021, and February 23rd, 2023 (Table S5) were only performed in the PubMed database because all included studies were identified in PubMed in the initial search.

**Table S5.** OVERVIEW OF LITERATURE SEARCHES.

| Database | Date of search | Number of records identified | Percentage of records that were identified in a previous search |
|---|---|---|---|
| **PubMed** | December 18th, 2020 | 10.815 | |
| **Embase** | December 18th, 2020 | 8938 | 29.0% |
| **Web of Science** | December 18th, 2020 | 8678 | 42.6% |
| **CINAHL** | December 18th, 2020 | 343 | 32.4% |
| **PubMed** | September 10th, 2021 | 772 | 1.0% |
| **PubMed** | February 8th, 2023 | 2356 | 36.2% |

# DATA SYNTHESIS

## GROUPING OF THE STUDIES

Based on previous topical reviews [14,103], we grouped the studies according to their primary outcome. A priori, we decided to categorize the outcomes in patient-reported outcomes (e.g. HRQoL, satisfaction or symptom burden), healthcare contacts (e.g. hospital admissions or outpatient contacts), patient-clinician communication, mortality and healthcare costs. However, based on the results, we categorized primary outcomes into six categories: 1. disease activity, 2. healthcare use and mortality, 3. mental well-being, 4. health-related quality of life, 5. self-efficacy, self-care, and daily functioning, and 6. other outcomes (e.g., feasibility, satisfaction, quality of care, or cost). Secondary outcomes were presented in a separate table for each study.

## STANDARDIZED METRICS AND TRANSFORMATION

The primary outcome was defined as the outcome used for deciding the overall result of the study. Consequently, the primary outcome was the outcome that served as the basis for the sample size calculation. If no power size calculation was presented, we used the protocol to decide the primary outcome. In case of no protocol, we used the primary outcome stated by the authors.

## PRIORITIZE RESULTS FOR SUMMARY AND SYNTHESIS

For randomized controlled trials, the overall result of the intervention was described as positive if the intervention group had a statistically significant better outcome of the intervention compared to the control group, and likewise negative if the intervention group had a statistically significant worse outcome compared to controls. If the estimates were similar or did not reach statistical significance, we concluded there was no effect of the intervention. If a study had more than one primary outcome and the results of these outcomes were heterogenous, we concluded that the intervention had mixed effects. For non-inferiority studies, we concluded non-inferiority if the difference between the intervention and control

group was below the non-inferior threshold. Pilot and feasibility studies were reported as feasible or not feasible based on the authors' conclusion of the study.

### Heterogeneity in reported effects

Because of variability in the patient populations, intervention types, outcomes assessed, and reporting of results, we deemed a meta-analysis unfeasible.

### Certainty of evidence

The risk of bias of each included RCT was assessed with the Cochrane Risk of Bias version 2 (CRoB2) [18]. The CRoB2 evaluates the risk of bias in five domains and overall. The risk of bias is based on judgement assisted by an algorithm. The five domains are the randomization process, deviations from the intended interventions, missing outcome data, measurement of the outcome, and selection of the reported result. The risk of bias can be judged as low, some concerns, or high.

### Data presentation methods

The key characteristics of the studies were recorded in a pre-designed text table in Word. If an intervention was reported in two or more papers, information on the first author and publication year from all papers were collapsed and reported under the primary publication. Furthermore, we extracted country, disease, age of the study participants (mean or interval), number of patients randomized, study design (all types of RCT), intervention type, main purpose (dialogue support, decision support, replace face-to-face visits, systematic PROM assessment alone), feedback (patient or healthcare professional with or without cut-off values), comparator (usual care, passive PROMs or other). Characteristics of the PROMs (type, validation status, and method of administration) were also extracted. In the case of two or more papers reporting the intervention, we used information from all papers to report the intervention and primary and secondary outcomes.

Based on the extracted information, we made a narrative synthesis presenting the characteristics of the studies, the PROM characteristics, and the outcomes. Furthermore, the data was presented in tables in summarized form in Table 2 and by study in Table 3 in the main paper.

# RESULTS

In the pre-designed table, we extracted information on characteristics of the included studies (Table S6), and the interventions (Table S7). We assessed risk of bias and determined primary outcomes (Table S8) as well as secondary outcomes of the included studies (Table S9).

**Table S6. CHARACTERISTICS OF INCLUDED STUDIES**

| Author | Year | Country | Disease | Age | No. of patients | Purpose | Study design | Comparator |
|---|---|---|---|---|---|---|---|---|
| Adams et al [1] | 2019 | USA | Diabetes | Mean 67 yrs. | 1270 | Systematic PROM assessment alone | Cluster-RCT, pragmatic | Usual care + three non-interactive educational calls |
| Bentley et al [2], Kargiannakis et al [3] | 2014 | UK | COPD | Mean 67 yrs. | 63 | Replace face- to-face visits | RCT, Pilot | Usual care |
| Berinstein et al [4] | 2021 | USA | IBD | Mean 47, 48 yrs. | 205 | Systematic PROM assessment and decision support | RCT | Usual care |
| Berkhof et al [5] | 2015 | The Netherlands | COPD | Mean 68 yrs. | 101 | Systematic PROM assessment alone | RCT, Pilot | Usual care |
| Bowles et al [6] | 2009 | USA | Heart disease and Diabetes | Mean 75 yrs. | 338 | Systematic PROM assessment alone | RCT, 3 arms | Usual care |
| Boyne et al [7-9], Gingele et al [10], Ramaekers et al [11] | 2009; 2012; 2013; 2013; 2019 | The Netherlands | Heart disease | Mean 71 yrs. | 382 | Systematic PROM assessment alone | RCT | Usual care |

| | | | | | | | | |
|---|---|---|---|---|---|---|---|---|
| Chaudhry et al [12], Jayaram et al [13] | 2010 | USA | Heart disease | Median 61 yrs. | 1653 | Systematic PROM assessment alone | RCT | Usual care |
| Cordova et al [14] | 2016 | USA | COPD | Mean 64, 63 yrs | 79 | Systematic PROM assessment alone | RCT | Passive PROM |
| Cross et al [15] | 2012 | USA | IBD | Mean 41 yrs. | 47 | Systematic PROM assessment alone | RCT | Usual care |
| Cross et al [16], Bilgrami et al [17,18], Quinn et al [19], Schliep et al [20] | 2019; 2020 | USA | IBD | Mean 39 yrs. | 348 | Systematic PROM assessment and decision support | RCT, 3 arms | Usual care |
| Dang et al [21,22] | 2017 | USA | Heart disease | Mean 55 yrs. | 61 | Systematic PROM assessment alone | RCT | Usual care |
| Dansky et al [23] | 2009 | USA | Heart disease | Mean 78 yrs. | 108 | Systematic PROM assessment alone | RCT | Usual care |
| De Jong et al [24,25] | 2017; 2020 | The Netherlands | IBD | Mean 44 yrs. | 909 | Systematic PROM assessment alone | RCT | Usual care |
| De Thurah et al [26], Skovsgaard et al [27] | 2018 | Denmark | RA | Mean 61, 62 yrs. | 294 | Replace face- to-face visits | RCT, 3 arms | Usual care |
| Del Hoyo et al [28,29] | 2018; 2019 | Spain | IBD | Median 39-41 yrs. | 63 | Replace face- to-face visits | RCT, 3 arms, Pilot | Usual care |

| | | | | | | | | |
|---|---|---|---|---|---|---|---|---|
| Elkjaer et al [30] | 2010 | Denmark, Ireland | IBD | Mean 41, 48 yrs. | 333 | Systematic PROM assessment and decision support | RCT | Usual care |
| Farmer et al [31], Velardo et al [32], Whelan et al [33] | 2017; 2019; 2020 | UK | COPD | Mean 70 yrs. | 166 | Systematic PROM assessment alone | RCT | Usual care |
| Frasure-Smith et al [34] | 1986 | Canada | Heart disease | Mean 58 yrs. | 461 | Systematic PROM assessment and decision support | RCT | Usual care |
| Frasure-Smith et al [35] | 1997 | Canada | Heart disease | Mean 59 yrs. | 1376 | Systematic PROM assessment alone | RCT | Usual care |
| GESICA Investigators [36] | 2005 | Argentina | Heart disease | Mean 65 yrs. | 1518 | Systematic PROM assessment and decision support | RCT | Usual care |
| Goldberg et al [37] | 2003 | USA | Heart disease | Mean 59 yrs. | 280 | Systematic PROM assessment alone | RCT | Usual outpatient care with instructions to record weight |
| Hernar et al [38] | 2021 | Norway | Diabetes | Mean 27 yrs. | 80 | Systematic PROM assessment and dialogue support | RCT, Pilot | Usual care |
| Hueppe et al [39] | 2014 | Germany | IBD | Mean 42 yrs. | 514 | Systematic PROM assessment and dialogue support | RCT | Usual care |

| | | | | | | | | |
|---|---|---|---|---|---|---|---|---|
| Huffstutter et al [40] | 2007 | USA | RA | Mean 60 yrs. | 901 | Systematic PROM assessment and dialogue support | RCT | Passive PROM |
| Johnson et al [41] | 2022 | USA | Heart disease | Mean 60 yrs. | 31 | Systematic PROM assessment alone | RCT, Pilot | Care as usual |
| Kronish et al [42], Ladapo et al [43] | 2020 | USA | Heart disease | Mean 66 yrs. | 1500 | Systematic PROM assessment and decision support | RCT, 3 arms | Usual care |
| Krum et al [44] | 2013 | Australia | Heart disease | Mean 73 yrs. | 405 | Systematic PROM assessment alone | Cluster-RCT | Usual care |
| Kuusalo et al [45] | 2020 | Finland | RA | Mean 59, 54 yrs. | 166 | Systematic PROM assessment alone | RCT | Usual care |
| Laurberg et al [46] | 2022 | Denmark | Diabetes | Mean 48 yrs. | 320 | Replace face- to-face visits | RCT, Noninferiority | Care as usual |
| Lee et al [47], Colls et al [48] | 2019; 2021 | USA | RA | Mean 52,2 years | 191 | Systematic PROM assessment alone | RCT | Care coordination alone |
| Lewis et al [49,50] | 2010; 2011 | UK | COPD | Mean 70, 67 yrs. | 40 | Systematic PROM assessment alone | RCT, Pilot | Usual care |
| McCombie et al [51] | 2020 | New Zealand | IBD | Mean 34, 35 yrs. | 107 | Replace face- to-face visits | RCT, Noninferiority | Usual care |

| | | | | | | | | |
|---|---|---|---|---|---|---|---|---|
| Ndosi et al [52] | 2016 | UK | RA | Mean 56, 54 yrs. | 132 | Systematic PROM assessment alone and decision support | RCT | Usual care |
| Nguyen et al [53] | 2009 | USA | COPD | Mean 64, 72 yrs. | 17 | Systematic PROM assessment alone | RCT, Pilot | Passive PROM |
| Paré et al [54] | 2013 | Canada | COPD | Mean 68, 69 yrs. | 120 | Systematic PROM assessment and decision support | RCT | Usual care |
| Park et al [55] | 2020 | Korea | COPD | Mean 68 yrs. | 42 | Systematic PROM assessment and dialogue support | RCT | Group education sessions and individual exercise in both groups |
| Pers et al [56], Bernard et al [57] | 2021 | France | RA | 18-75 yrs. | 94 | Systematic PROM assessment and decision support | RCT | Usual care |
| Piette et al [58] | 2001 | USA | Diabetes | Mean 60-61 yrs. | 292 | Systematic PROM assessment and decision support | RCT | Usual care |
| Piette et al [59,60] | 2000 | USA | Diabetes | 18-75 yrs. | 280 | Systematic PROM assessment and decision support | RCT | Usual care |
| Pinnock et al [61], Stoddart et al [62] | 2013 | Scotland | COPD | Mean 68-69 yrs. | 256 | Systematic PROM assessment and decision support | RCT | Usual care |

| | | | | | | | | |
|---|---|---|---|---|---|---|---|---|
| Pouwer et al [63] | 2001 | the Netherlands | Diabetes | Mean 53, 54 yrs. | 400 | Systematic PROM assessment and dialogue support | RCT | Usual care |
| Pouwer et al [64] | 2011 | the Netherlands | Diabetes | Mean 52, 55 yrs. | 223 | Systematic PROM assessment and decision support | RCT | Usual care |
| Rassouli et al [65] | 2021 | Switzerland and Germany | COPD | Median 67 yrs. | 168 | Systematic PROM assessment and decision support | RCT | Usual care |
| Schwarz et al [66] | 2008 | USA | Heart disease | Mean 77, 79 yrs. | 102 | Systematic PROM assessment alone | RCT, Pilot | Usual care |
| Scollan-Koliopoulos et al [67] | 2012 | USA | Diabetes | 22-89 yrs. | 103 | Systematic PROM assessment and dialogue support | RCT | Usual care |
| Sethares et al [68] | 2004 | USA | Heart disease | Mean 76, 77 yrs. | 70 | Systematic PROM assessment alone | RCT | Usual care |
| Shara et al [69] | 2022 | USA | Heart disease | Mean 54 yrs. | 60 | Systematic PROM assessment alone | RCT, Pilot | Care as usual |
| Sink et al [70] | 2020 | USA | COPD | Mean 60, 62 yrs. | 168 | Systematic PROM assessment and decision support | RCT | Passive PROM |
| Slok et al [71] | 2016 | the Netherlands | COPD | Mean 62, 66 yrs. | 357 | Systematic PROM assessment and dialogue support | Cluster-RCT | Usual care |

| | | | | | | | | |
|---|---|---|---|---|---|---|---|---|
| Soran et al [72] | 2008 | USA | Heart disease | Mean 73, 77 yrs. | 315 | Systematic PROM assessment and decision support | RCT | Usual care |
| Spaeder et al [73] | 2006 | USA | Heart disease | Mean 53, 56 yrs. | 49 | Systematic PROM assessment and decision support | RCT | Usual care |
| Subramanian et al [74], Keeffe et al [75] | 2004; 2005 | USA | Heart disease | Mean 69 yrs. | 720 | Systematic PROM assessment and decision support | Cluster-RCT | Usual care |
| van Dijk-de Vries et al [76] | 2015 | the Netherlands | Diabetes | Mean 64, 65 yrs. | 264 | Systematic PROM assessment alone | Cluster-RCT | Usual care |
| Vo et al [77] | 2019 | USA | Diabetes | Mean 61 yrs. | 1276 | Systematic PROM assessment and dialogue support | RCT | Usual care |
| Zakrisson et al [78] | 2020 | Sweden | COPD | Mean 71, 72 yrs. | 220 | Systematic PROM assessment and dialogue support | RCT | Usual care |
| Östlund et al [79] | 2021 | Sweden | IBD | Median 35, 37 yrs. | 200 | Systematic PROM assessment and decision support | RCT | Usual care |

Abbreviations: PROM=patient-reported outcome measure, COPD=chronic obstructive pulmonary disease, IBD=inflammatory bowel disease, RA=rheumatoid arthritis, RCT=randomised controlled trial

**Table S7.** CHARACTERISTICS OF THE INTERVENTIONS IN THE INCLUDED STUDIES.

| Author | Year | Disease | Intervention | PROM intervention | PROM intervention validation | Methods for administration of PROM |
|---|---|---|---|---|---|---|
| Adams et al [1] | 2019 | Diabetes | Three interactive voice response calls over 6 months collecting information on symptom relief, medication use, titration, discontinuation, and side effects. | Unspecified questions about symptoms and medication | Unspecified | Telephone |
| Bentley et al [2], Kargiannakis et al [3] | 2014 | COPD | Daily self-report of symptoms and monitoring of vital signs on a device to replace home visits. | Specified symptom questions | Specified | Device |
| Berinstein et al [4] | 2021 | IBD | Proactive symptom monitoring and care coordinator triggered algorithms monthly, after hospitalization, or initiated by the patient. | Validated symptom questionnaire | Validated | Unknown |
| Berkhof et al [5] | 2015 | COPD | Structured phone call by nurse every 2 weeks for 6 months. Brief introductory conversation followed by a short, validated health status questionnaire. | Validated symptom questionnaire | Validated | Telephone |
| Bowles et al [6] | 2009 | Heart disease and Diabetes | Telephone group: Standardized telephone interview about symptoms by a nurse; Telemonitoring group: Physiological monitoring of vital signs and videoconferencing. | Specified questions about symptoms, medications, and health behavior | Specified | Telephone |
| Boyne et al [7-9], Gingele et al [10], Ramaekers et al [11] | 2009; 2012; 2013; 2013; 2019 | Heart disease | Daily pre-set dialogues about symptoms, knowledge and behavior via a device connected to the telephone. | Unspecified symptom questions | Unspecified | Device |
| Chaudhry et al [12], Jayaram et al [13] | 2010 | Heart disease | Daily automated symptom and weight questions by telephone and prespecified variance alerts triggered clinical contacts. | Unspecified questions about symptoms and general health, and a validated depression symptom | Unspecified | Telephone |

| | | | | | | |
|---|---|---|---|---|---|---|
| | | | Depression symptoms monitored monthly. | questionnaire assessment | | |
| Cordova et al [14] | 2016 | COPD | Daily patient-reported symptoms and functional status via an electronic diary. Daily peak flow. | Unspecified symptom questions and validated functional status | Unspecified | Device |
| Cross et al [15] | 2012 | IBD | Weekly monitoring of symptoms, side effects, medication adherence and weight using a netbook computer and an electronic weight scale. | Unspecified questions about symptoms and medication | Unspecified | Laptop |
| Cross et al [16], Bilgrami et al [17,18], Quinn et al [19], Schliep et al [20] | 2019; 2020 | IBD | Monitoring of symptoms and side effects using a mobile phone weekly or every other week. Both arms also included educational text message tips. | Validated disease activity questionnaire and unspecified questions about medication | Validated | Web page |
| Dang et al [21,22] | 2017 | Heart disease | Daily monitoring of weight and symptoms vi mobile phone. | Specified symptom questions | Specified | SMS |
| Dansky et al [23] | 2009 | Heart disease | Monitoring of symptoms, self-care practices, and medication compliance via the Health Buddy device. | Unspecified symptom questions | Unspecified | Device |
| De Jong et al [24,25] | 2017; 2020 | IBD | Monthly monitoring of disease activity, medication use, treatment adherence, treatment satisfaction, and side effects. Adjusts monitoring frequency to treatment activity. Includes personal care plan and e-learning. | Validated disease activity questionnaire and unspecified questions about symptoms and medication | Validated | Web page |
| De Thurah et al [26], Skovsgaard et al [27] | 2018 | RA | Quarterly patient-reported symptom questionnaire assessment and telephone consultation by a doctor or a nurse to replace physical visits. | Validated questionnaire on disease activity | Validated | Web page |

| | | | | | | |
|---|---|---|---|---|---|---|
| Del Hoyo et al [28,29] | 2018; 2019 | IBD | Telemonitoring group: App-based self-report of symptoms and adverse effects with action plans; Telephone group: structured interviews on symptoms carried out by a nurse. | Unspecified questions about symptoms and adverse effects | Unspecified | Mobile app |
| Elkjaer et al [30] | 2010 | IBD | Web-based report of PROMs on disease activity and QoL weekly in case of relapse, otherwise monthly. | Validated questionnaire on disease activity | Validated | Web page |
| Farmer et al [31], Velardo et al [32], Whelan et al [33] | 2017; 2019; 2020 | COPD | Daily self-report of symptoms, oxygen levels, and heart rate. Alerts by individual thresholds after the run-in period. | Specified questions about symptoms and medication, and validated questionnaire on mood | Specified | Tablet app |
| Frasure-Smith et al [34] | 1986 | Heart disease | Monthly structured telephone interviews about the psychosocial burden and nursing psychosocial support. | Validated general health questionnaire | Validated | Telephone |
| Frasure-Smith et al [35] | 1997 | Heart disease | Monthly structured telephone interviews about the psychosocial burden and nursing psychosocial support. | Validated general health questionnaire | Validated | Telephone |
| GESICA Investigators [36] | 2005 | Heart disease | Telephone follow-up as needed by a nurse to educate and monitor the patient about diet, medication, symptoms, hydrosaline retention, and daily physical activity. | Specified questions about symptoms, medication, and physical activity | Specified | Telephone |
| Goldberg et al [37] | 2003 | Heart disease | Automated telephone-response system with personalized questions about symptoms and weight. | Unspecified symptom questions | Unspecified | Telephone |

| | | | | | | |
|---|---|---|---|---|---|---|
| Hernar et al [38] | 2021 | Diabetes | Waiting room PROMs on diabetes distress before two annual consultations and referral to nurse clinic upon high distress scores. | Validated diabetes distress questionnaire | Validated | Tablet app |
| Hueppe et al [39] | 2014 | IBD | Patient activation through advice feedback on patient-reported symptoms and psychological problems from a postal questionnaire. | Specified symptom questions | Specified | Paper |
| Huffstutter et al [40] | 2007 | RA | Waiting room touch screen on patient-reported symptoms, disability, HRQoL, and medication use visualized for use at clinic visits. | Validated symptom questionnaire, and physician and patient global assessment | Validated | Tablet app |
| Johnson et al [41] | 2022 | Heart disease | Daily patient-report of symptoms, feedback, and educational videos | Unspecified symptom questions | Unspecified | Mobile app |
| Kronish et al [42], Ladapo et al [43] | 2020 | Heart disease | Treatment group: Depression symptom questionnaire assessment, notification of primary care provider, and stepped treatment plan; Notify only group: Depression symptom questionnaire assessment and notification of primary care provider. | Validated depression questionnaire | Validated | Unknown |
| Krum et al [44] | 2013 | Heart disease | Telemedicine system with monthly and as-needed self-report on disease burden, disease management, and health behavior. | Specified questions about disease burden, and health behavior | Specified | Telephone |
| Kuusalo et al [45] | 2020 | RA | Self-report of symptoms and medications through 13 SMS over 24 weeks. | Specified questions about symptoms and medication | Specified | SMS |

| | | | | | | |
|---|---|---|---|---|---|---|
| Laurberg et al [46] | 2022 | Diabetes | PRO questionnaire every 4th month before visit | Validated questionnaires on HRQoL and diabetes distress, and specified questions about general health | Validated | Web page |
| Lee et al [47], Colls et al [48] | 2019; 2021 | RA | Smartphone app with care coordination to monitor daily electronic PROMs on physical function, disease activity, pain interference, fatigue, sleep disturbance, and depression. | Validated questionnaire on symptoms and disease activity | Validated | Mobile app |
| Lewis et al [49,50] | 2010; 2011 | COPD | Telemonitoring with self-report of symptoms, temperature. and oxygen levels twice daily. | Specified symptom questions | Specified | Device |
| McCombie et al [51] | 2020 | IBD | Quarterly and as-needed app-based self-reporting of symptoms and fecal calprotectin. | Validated questionnaire on disease activity | Validated | Mobile app |
| Ndosi et al [52] | 2016 | RA | Waiting room assessment of educational needs based on answers to questions about managing symptoms, mental health, treatments, daily functioning, self-help measures, and support | Validated questionnaire on educational needs | Validated | Unknown |
| Nguyen et al [53] | 2009 | COPD | Daily self-report of symptoms and exercise in a cell phone-based system with text-message feedback and telephone contact as needed. | Specified questions about exercise | Specified | Mobile app |
| Paré et al [54] | 2013 | COPD | Self-report of symptoms and medication use on a tablet, automated self-management program with learning modules. | Unspecified symptom questions | Unspecified | Device |
| Park et al [55] | 2020 | COPD | Self-report of symptoms, medication use and physical activity in a smartphone app. | Validated symptom questionnaire | Validated | Mobile app |

| | | | | | | |
|---|---|---|---|---|---|---|
| Pers et al [56], Bernard et al [57] | 2021 | RA | Weekly self-report of symptoms and handgrip strength in a smartphone app. | Validated symptom questionnaire and handgrip strength | Validated | Mobile app |
| Piette et al [58] | 2001 | Diabetes | Automated phone calls with self-care education and assessment of blood glucose, self-care, and symptoms. | Unspecified questions about symptoms and self-care | Unspecified | Telephone |
| Piette et al [59,60] | 2000 | Diabetes | Biweekly automated phone calls with self-care education and assessment of blood glucose, self-care, and symptoms. | Unspecified questions about symptoms and self- care | Unspecified | Telephone |
| Pinnock et al [61], Stoddart et al [62] | 2013 | COPD | Daily self-report of symptoms, use of treatment, and oxygen saturation using a touch screen. | Specified questions about symptoms and medication | Specified | Device |
| Pouwer et al [63] | 2001 | Diabetes | Psychological well-being questionnaire assessment and counselling in addition to usual quarterly clinic visits. | Validated well-being questionnaire | Validated | Web page |
| Pouwer et al [64] | 2011 | Diabetes | Diagnostic interview for mood and anxiety disorders. A diagnosis was followed by treatment advice to the general practitioner and diabetes specialist, whereas the absence of diagnosis was followed by self-management support. | Validated diagnostic interview | Validated | Interview |
| Rassouli et al [65] | 2021 | COPD | Daily questions focused on recognizing acute exacerbation in an online platform available from a computer or smartphone. | Specified symptom questions | Specified | Web page |
| Schwarz et al [66] | 2008 | Heart disease | Daily self-report of weight, symptoms, medication, and health behavior transferred via a telephone line to a central server. | Unspecified questions about symptoms, medication, and health behavior | Unspecified | Device |

| | | | | | | |
|---|---|---|---|---|---|---|
| Scollan-Koliopoulos et al [67] | 2012 | Diabetes | Brief needs-based assessment of emotional reactions to diabetes as a supplement to the clinic visit. | Specified questions about emotional reactions and self-care behavior | Specified | Interview |
| Sethares et al [68] | 2004 | Heart disease | Messages tailored to perceived benefits and barriers of self-care during and two times after hospitalization. | Validated health belief scale | Validated | Interview/consultati on |
| Shara et al [69] | 2022 | Heart disease | Daily patient-report of self-care, medication, and symptoms through voice activated technology for 90 days | Specified questions about symptoms, medication, and self-care | Specified | Device |
| Sink et al [70] | 2020 | COPD | Daily automated phone calls or text messages on breathing status with provider counselling in case of worsening. | One specified question on breathing status | Specified | Telephone |
| Slok et al [71] | 2016 | COPD | Measurement and visualization of self-reported symptoms and physical activity in at least four consultations during the 18-month follow-up. Daily self-report of weight and symptoms in a telephone-connected monitor and personalized benchmarks. | Validated questionnaire on symptoms, functional status and mental symptoms | Validated | Web page |
| Soran et al [72] | 2008 | Heart disease | | Unspecified symptom questions | Unspecified | Device |
| Spaeder et al [73] | 2006 | Heart disease | Telephone-based automated daily questions about physiological parameters, symptoms, medication use and side effects during carvedilol titration. | Unspecified symptom questions and medication | Unspecified | Telephone |

| | | | | | | |
|---|---|---|---|---|---|---|
| Subramanian et al [74], Keeffe et al [75] | 2004; 2005 | Heart disease | Patient-reported symptoms from questionnaires mailed to patients within 2 weeks of scheduled outpatient visits were used to generate medication suggestions. | Validated symptom questionnaire | Validated | Paper |
| van Dijk-de Vries et al [76] | 2015 | Diabetes | Detection and follow-up on mental health symptoms via SMS with self-management support in case of mental distress. | Specified distress scale and validated mental symptom questionnaire | Specified | Telephone |
| Vo et al [77] | 2019 | Diabetes | Patient-reported prioritization of concerns before clinic visits through a website. | Specified questions about diabetes- related concerns and medication | Specified | Web page |
| Zakrisson et al [78] | 2020 | COPD | Patient-reported needs using a paper questionnaire and priorities before clinic visit. | Specified questionnaire on psychosocial needs and priorities | Specified | Paper |
| Östlund et al [79] | 2021 | IBD | Self-report of symptoms, disease activity, and fecal calprotectin upon suspected flare or remission. | Validated symptom questionnaire | Validated | Mobile app |

Abbreviations: PROM=patient-reported outcome measure, COPD=chronic obstructive pulmonary disease, IBD=inflammatory bowel disease, RA=rheumatoid arthritis, RCT=randomised controlled trial, App=application, SMS=short message service

Table S8. RISK OF BIAS AND PRIMARY OUTCOMES OF INCLUDED STUDIES.

| Author | Year | Disease | Purpose | PROM intervention | RoB | Primary outcome (effect) |
|---|---|---|---|---|---|---|
| Adams et al [1] | 2019 | Diabetes | Assessment alone | Unspecified questions about symptoms and medication | Low | QoL (no effect) |
| Bentley et al [2], Kargiannakis et al [3] | 2014 | COPD | Replace face-to-face visits | Specified symptom questions | Some concerns | Hospital readmissions and HRQoL (not feasible) |
| Berinstein et al [4] | 2021 | IBD | Decision support | Validated symptom questionnaire | High | IBD-symptom scores and healthcare charges (mixed: Improved IBD- symptom scores but no effect on healthcare charges) |
| Berkhof et al [5] | 2015 | COPD | Assessment alone | Validated symptom questionnaire | High | QoL (mixed: No effect on total score, but a statistically significant deterioration in the symptom domain) |
| Bowles et al [6] | 2009 | Heart disease and Diabetes | Assessment alone | Specified questions about symptoms, medications, and health behavior | High | Readmission at 60 days (negative) |
| Boyne et al [7-9], Gingele et al [10], Ramaekers et al [11] | 2009; 2012; 2013; 2013; 2019 | Heart disease | Assessment alone | Unspecified symptom questions | High | Time to first HF hospitalization (no effect) |
| Chaudhry et al [12], Jayaram et al [13] | 2010 | Heart disease | Assessment alone | Unspecified questions about symptoms and general health, and a validated depression symptom questionnaire assessment | Low | Composite outcome of admission and death (no effect) |
| Cordova et al [14] | 2016 | COPD | Assessment alone | Unspecified symptom questions and validated functional status | Some concerns | Composite outcome of admission and death (no effect) |

| | | | | | | |
|---|---|---|---|---|---|---|
| Cross et al [15] | 2012 | IBD | Assessment alone | Unspecified questions about symptoms and medication | Low | Disease activity, QoL, medication adherence (no effect) |
| Cross et al [16], Bilgrami et al [17,18], Quinn et al [19], Schliep et al [20] | 2019; 2020 | IBD | Decision support | Validated disease activity questionnaire and unspecified questions about medication | Some concerns | Disease activity and disease specific QoL (no effect) |
| Dang et al [21,22] | 2017 | Heart disease | Assessment alone | Specified symptom questions | Low | Self-Efficacy for managing chronic disease (positive) |
| Dansky et al [23] | 2009 | Heart disease | Assessment alone | Unspecified symptom questions | High | Health and functional status (positive) |
| De Jong et al [24,25] | 2017; 2020 | IBD | Assessment alone | Validated disease activity questionnaire and unspecified questions about symptoms and medication | High | Outpatient visits, quality of care (mixed: Reduced outpatient visits and no effect on quality of care) |
| De Thurah et al [26], Skovsgaard et al [27] | 2018 | RA | Replace face-to-face visits | Validated questionnaire on disease activity | Some concerns | Disease activity (noninferiority in both intervention arms) |
| Del Hoyo et al [28,29] | 2018; 2019 | IBD | Replace face-to-face visits | Unspecified questions about symptoms and adverse effects | Some concerns | Clinical remission (no effect) |
| Elkjaer et al [30] | 2010 | IBD | Decision support | Validated questionnaire on disease activity | High | Feasibility (feasible) |
| Farmer et al [31], Velardo et al [32], Whelan et al [33] | 2017; 2019; 2020 | COPD | Assessment alone | Specified questions about symptoms and medication, and validated questionnaire on mood | High | QoL (no effect) |
| Frasure-Smith et al [34] | 1986 | Heart disease | Decision support | Validated general health questionnaire | Some concerns | Admissions and mortality (mixed: Mortality reduction and no effect on admissions) |
| Frasure-Smith et al [35] | 1997 | Heart disease | Assessment alone | Validated general health questionnaire | High | Cardiac mortality (no effect) |

| | | | | | | |
|---|---|---|---|---|---|---|
| GESICA Investigators [36] | 2005 | Heart disease | Decision support | Specified questions about symptoms, medication, and physical activity | Some concerns | Composite outcome of hospital admission and death (positive) |
| Goldberg et al [37] | 2003 | Heart disease | Assessment alone | Unspecified symptom questions | Some concerns | Readmission (no effect) |
| Hernar et al [38] | 2021 | Diabetes | Dialogue support | Validated diabetes distress questionnaire | Some concerns | Feasibility (feasible) |
| Hueppe et al [39] | 2014 | IBD | Dialogue support | Specified symptom questions | High | HRQoL, social participation, and disability days (positive) |
| Huffstutter et al [40] | 2007 | RA | Dialogue support | Validated symptom questionnaire, and physician and patient global assessment | Some concerns | Patient satisfaction and physician-patient interaction (mixed: No effect on patient satisfaction but improved interaction reported by physicians) |
| Johnson et al [41] | 2022 | Heart disease | Assessment alone | Unspecified symptom questions | High | Feasibility (feasible) |
| Kronish et al [42], Ladapo et al [43] | 2020 | Heart disease | Decision support | Validated depression questionnaire | Some concerns | Quality-adjusted life years (no effect) |
| Krum et al [44] | 2013 | Heart disease | Assessment alone | Specified questions about disease burden, and health behavior | Some concerns | Packer clinical composite score (no effect) |
| Kuusalo et al [45] | 2020 | RA | Assessment alone | Specified questions about symptoms and medication | High | Remission (no effect) |
| Laurberg et al [46] | 2022 | Diabetes | Replace face-to-face visits | Validated questionnaires on HRQoL and diabetes distress, and specified questions about general health | Low | HbA1c (non-inferiority) |
| Lee et al [47], Colls et al [48] | 2019; 2021 | RA | Assessment alone | Validated questionnaire on symptoms and disease activity | Some concerns | Treatment satisfaction (no effect) |
| Lewis et al [49,50] | 2010; 2011 | COPD | Assessment alone | Specified symptom questions | Some concerns | Feasibility (feasible) |

| | | | | | | |
|---|---|---|---|---|---|---|
| McCombie et al [51] | 2020 | IBD | Replace face-to-face visits | Validated questionnaire on disease activity | High | HRQoL & symptoms (non-inferiority) |
| Ndosi et al [52] | 2016 | RA | Decision support | Validated questionnaire on educational needs | High | Self-efficacy (positive) |
| Nguyen et al [53] | 2009 | COPD | Assessment alone | Specified questions about exercise | Some concerns | Feasibility (feasible) |
| Paré et al [54] | 2013 | COPD | Decision support | Unspecified symptom questions | High | Healthcare costs (positive) |
| Park et al [55] | 2020 | COPD | Dialogue support | Validated symptom questionnaire | High | Self-care behavior (positive) |
| Pers et al [56], Bernard et al [57] | 2021 | RA | Decision support | Validated symptom questionnaire and handgrip strength | High | Physical visits to the hospital (positive) |
| Piette et al [58] | 2001 | Diabetes | Decision support | Unspecified questions about symptoms and self-care | High | HbA1c (no effect) |
| Piette et al [59,60] | 2000 | Diabetes | Decision support | Unspecified questions about symptoms and self-care | Some concerns | HbA1c (no effect) |
| Pinnock et al [61], Stoddart et al [62] | 2013 | COPD | Decision support | Specified questions about symptoms and medication | Some concerns | Time to admission (no effect) |
| Pouwer et al [63] | 2001 | Diabetes | Dialogue support | Validated well-being questionnaire | High | Psychological well-being, Hba1c, and quality of care (mixed: Positive effect on psychological well-being, no effect on Hba1c, or quality of care) |
| Pouwer et al [64] | 2011 | Diabetes | Decision support | Validated diagnostic interview | Low | Depression score (no effect) |
| Rassouli et al [65] | 2021 | COPD | Decision support | Specified symptom questions | High | COPD assessment test (positive) |
| Schwarz et al [66] | 2008 | Heart disease | Assessment alone | Unspecified questions about symptoms, medication, and health behavior | High | 3 months readmission (not feasible) |

| | | | | | | |
|---|---|---|---|---|---|---|
| Scollan- Koliopoulos et al [67] | 2012 | Diabetes | Dialogue support | Specified questions about emotional reactions and self-care behavior | Some concerns | Depression (no group comparison) |
| Sethares et al [68] | 2004 | Heart disease | Assessment alone | Validated health belief scale | Low | Readmission rate (no effect) |
| Shara et al [69] | 2022 | Heart disease | Assessment alone | Specified questions about symptoms, medication, and self-care | High | Hospitalization (negative) |
| Sink et al [70] | 2020 | COPD | Decision support | One specified question on breathing status | High | Time to hospitalization (positive) |
| Slok et al [71] | 2016 | COPD | Dialogue support | Validated questionnaire on symptoms, functional status and mental symptoms | Some concerns | QoL (positive) |
| Soran et al [72] | 2008 | Heart disease | Decision support | Unspecified symptom questions | Some concerns | Rehospitalization or mortality (no effect) |
| Spaeder et al [73] | 2006 | Heart disease | Decision support | Unspecified symptom questions and medication | High | Titration time (positive) |
| Subramanian et al [74], Keeffe et al [75] | 2004; 2005 | Heart disease | Decision support | Validated symptom questionnaire | High | Physician adherence to treatment suggestions (no effect) |
| van Dijk-de Vries et al [76] | 2015 | Diabetes | Assessment alone | Specified distress scale and validated mental symptom questionnaire | High | Daily functioning (no effect) |
| Vo et al [77] | 2019 | Diabetes | Dialogue support | Specified questions about diabetes-related concerns and medication | High | HbA1c (no effect) |
| Zakrisson et al [78] | 2020 | COPD | Dialogue support | Specified questionnaire on psychosocial needs and priorities | Some concerns | Quality of care (no group comparison) |
| Östlund et al [79] | 2021 | IBD | Decision support | Validated symptom questionnaire | Some concerns | Acceptance and adherence (feasible) |

Abbreviations: PROM=patient-reported outcome measure, COPD=chronic obstructive pulmonary disease, IBD=inflammatory bowel disease, RA=rheumatoid arthritis, QoL=quality of life, HRQoL=health related quality of life, HbA1c=Hemoglobin A1c, RCT=randomized controlled trial, HF: heart failure

**Table S9**. SECONDARY OUTCOMES OF INCLUDED STUDIES.

| Author | Year | Disease | Intervention | Secondary outcomes (effect) |
|---|---|---|---|---|
| Adams et al [1] | 2019 | Diabetes | Three interactive voice response calls over 6 months collecting information on symptom relief, medication use, titration, discontinuation, and side effects. | Clinical care (no effect)<br>Physical Symptoms (no effect on lower extremity functioning)<br>Depression (no effect) Medication (no effect) Sleep (no effect)<br>Communication (no effect) |
| Bentley et al [2], Kargiannakis et al [3] | 2014 | COPD | Daily self-report of symptoms and monitoring of vital signs on device to replace home visits | Mortality (assessed but not estimated)<br>Hospitalizations, emergency department visits and community nurse visits (assessed but not estimated)<br>QoL (assessed but not estimated)<br>Cost (Higher costs) |
| Berinstein et al [4] | 2021 | IBD | Proactive symptom monitoring and care coordinator triggered algorithms monthly, after hospitalization, and patient-initiated | ED visits (no effect)<br>Use of medication (no change) |
| Berkhof et al [5] | 2015 | COPD | Structured phone call by nurse every 2 weeks for 6 months. Brief introductory conversation followed by a short, validated health status questionnaire | QoL (descriptive results in favor of control group)<br>Physical symptoms (deterioration) |
| Bowles et al [6] | 2009 | Heart disease & Diabetes | Telephone group: Standardized telephone interview about symptoms by nurse, Telemonitoring group: Physiological monitoring of vital signs and videoconferencing | ED visits (no effect)<br>Physical symptoms (no effect on severity or health status)<br>Depression (no effect)<br>Knowledge, behavior (no effect) |
| Boyne et al [7-9], Gingele et al [10], Ramaekers et al [11] | 2009; 2012; 2013; 2013; 2019 | Heart disease | Daily pre-set dialogues about symptoms, knowledge, and behavior | Mortality (no effect)<br>Hospitalizations, hospitalization days, specialist visits (no effect), nurse contacts (fewer)<br>QoL (no effect on EQ-5D)<br>Physical symptoms (Increased metabolic equivalent scores)<br>Knowledge (improved)<br>Selfcare, Self-efficacy (improved) Cost (unclear) |

| | | | | |
|---|---|---|---|---|
| Chaudhry et al [12], Jayaram et al [13] | 2010 | Heart disease | Daily automated symptom and weight questions by telephone and prespecified variance alerts triggered clinical contacts. Depression symptoms monitored monthly. | Mortality (no effect) Readmissions, days in hospital (no effect)<br>Physical symptoms, health status (improved) |
| Cordova et al [14] | 2016 | COPD | Daily patient-reported symptoms and functional status via an electronic diary. Daily peak flow. | Mortality (no effect)<br>Clinical parameters (Improved peak flow)<br>QoL (No effect on main SF-36 score)<br>Physical symptoms (improved) |
| Cross et al [15] | 2012 | IBD | Weekly monitoring of symptoms, side effects, medication adherence and weight. | |
| Cross et al [16], Bilgrami et al [17,18], Quinn et al [19], Schliep et al [20] | 2019; 2020 | IBD | Monitoring of symptoms and side effects through the TELE-IBD system weekly (arm 1) or every other week (arm 2).<br>Both arms also included educational text message tips. | Hospitalization (reduced), electronic encounters (increased)<br>Clinical parameters (increased diagnostic tests)<br>QoL (no effect)<br>Physical symptoms (disease activity, subgroup analysis in favor of controls) Depression (no effect)<br>Patient activation (no effect)<br>Self-efficacy (no effect) |
| Dang et al [21,22] | 2017 | Heart disease | Daily monitoring of weight and symptoms | QoL (improved)<br>Health distress (improved) |
| Dansky et al [23] | 2009 | Heart disease | Monitoring of symptoms, self-care practices, and medication compliance via the Health Buddy device | Hospitalization (fewer)<br>Clinical parameters (descriptive results of respiratory status in favor of intervention)<br>Daily living (descriptive results in favor of intervention) |

| | | | | |
|---|---|---|---|---|
| De Jong et al [24,25] | 2017; 2020 | IBD | Monthly monitoring of disease activity, medication use, treatment adherence, treatment satisfaction, and side-effects. Adjusts monitoring frequency to treatment activity. Includes personal care plan and e-learning. | Hospitalization, gastroenterologist consultations (fewer), nurse consultations (increased), ED visits (no effect)<br>Clinical parameters (no effect on corticosteroid courses or surgeries) QoL (no effect)<br>Physical symptoms (no effect on flares) Depression (no effect)<br>Use of medication (improved) Knowledge (no effect)<br>Self-efficacy (no effect)<br>Cost (saving and probably cost-effective) |
| De Thurah et al [26], Skovsgaard et al [27] | 2018 | RA | Quarterly patient-reported symptom assessment and telephone consultation to replace physical visits by 1. Doctor or<br>2. Nurse | Visits (descriptive results in favor of intervention)<br>QoL (no effect on 5Q-5D) Self-efficacy (no effect)<br>Level of function (no effect on HAQ) |
| Del Hoyo et al [28,29] | 2018; 2019 | IBD | 1: TECCU app-based self-report of symptoms and adverse effects with action plans, 2: Telephone-based nursing care | Outpatient visits (descriptive results in favor of intervention)<br>Clinical parameters (no effect on fecal calprotectin)<br>QoL (no effect on ED-5Q, IBDQ-9)<br>Use of medication (improved adherence) Work productivity, social activities (no effect)<br>Cost (no effect) |
| Elkjaer et al [30] | 2010 | IBD | Web-based e-health, patient input in case of disease activity | ED visits, routine visits (reduced) Clinical parameters (descriptive results on fecal calprotectin)<br>QoL (improved)<br>Physical symptoms (reduced time to remission)<br>Depression (increased)<br>Use of medication (improved adherence and knowledge)<br>Cost (saving) |

| | | | | |
|---|---|---|---|---|
| Farmer et al [31], Velardo et al [32], Whelan et al [33] | 2017; 2019; 2020 | COPD | Daily self-report of symptoms, oxygen levels, and heart rate. Alerts by individual thresholds after run-in period | Mortality (no effect)<br>Clinical parameters (no effect on lung function or exacerbations)<br>GP contacts, hospitalizations (no effect), nurse contacts (fewer)<br>QoL (Improved EQ-5D) Depression and anxiety (no effect) Medication adherence (no effect)<br>Beliefs (no effect)<br>Smoking (no effect) |
| Frasure-Smith et al [34] | 1986 | Heart disease | Monthly structured telephone interviews about psychosocial burden and nursing psychosocial support | Stress (reduced) |
| Frasure-Smith et al [35] | 1997 | Heart disease | Monthly structured telephone interviews about psychosocial burden and nursing psychosocial support | Mortality (no effect on overall mortality but Indication of increased mortality in women)<br>Clinical parameters (no effect on re- infarctions)<br>Hospitalizations (no effect)<br>Depression, anxiety (no effect) |
| GESICA Investigators [36] | 2005 | Heart disease | Telephone follow-up as needed by nurse to educate and monitor the patient about diet, medication, symptoms, hydrosaline retention, and daily physical activity. | Mortality (no effect) Hospitalizations (fewer) QoL (improved) Medication (increased) |
| Goldberg et al [37] | 2003 | Heart disease | Automated telephone-response system with personalized questions about symptoms and weight | Mortality (positive)<br>Hospitalization, ED visits (no effect) QoL (no effect) |
| Hernar et al [38] | 2021 | Diabetes | Waiting room PROMs before two annual consultations and referral to nurse clinic upon high distress scores | Clinical parameters (no effect on HbA1c) Well-being (no effect)<br>Perceived competence (no effect) |
| Hueppe et al [39] | 2014 | IBD | Patient activation through advice feedback on patient-reported symptoms | Hospitalization (no effect), outpatient visits (fewer), allied health professional visits, rehabilitation (no effect)<br>Physical symptoms (no effect) Medication use (no effect)<br>Self-management skills (no effect) |
| Huffstutter et al [40] | 2007 | RA | Patient-reported symptoms, disability, HRQoL, and use of medication visualized for use in clinic visits | |

| | | | | |
|---|---|---|---|---|
| Johnson et al [41] | 2022 | Heart disease | Daily patient-report of symptoms, feedback, and educational videos | QoL (no effect)<br>Readmission (no effect) Death (no effect) |
| Kronish et al [42], Ladapo et al [43] | 2020 | Heart disease | 1. Depression symptom assessment, notification of primary care provider, and stepped treatment plan. 2. Depression symptom assessment and notification of primary care provider | Mortality (no effect)<br>Clinical parameters (no effect on utility) QoL (no effect)<br>Depression (no effect)<br>Patient-reported harms (no effect) |
| Krum et al [44] | 2013 | Heart disease | Monthly and as-needed self-report on disease burden, disease management, and health behavior | Mortality (no effect)<br>Hospitalization (fewer all-cause, no effect on heart failure hospitalization) |
| Kuusalo et al [45] | 2020 | RA | Self-report of symptoms and medications through 13 SMS-es over 24 weeks | Nurse telephone contacts (increased), unscheduled nurses' visits, physician telephone contacts, unscheduled physician visits (no effect)<br>Clinical parameters (no effect on DAS28) QoL (no effect)<br>Use of medication (no change), confidence of treatment (no effect) |
| Laurberg et al [46] | 2022 | Diabetes | PRO questionnaire every 4th month before visit | Mean lipid and blood pressure (non- inferior)<br>Total visits (increase)<br>Face-to-face visits (reduction) Participation (improved)<br>Well-being (WHO-5, improved) Diabetes distress (PAID, reduction)<br>Cancellation of visits (reduction) |
| Lee et al [47], Colls et al [48] | 2019; 2021 | RA | Smartphone app with care coordination to monitor daily electronic PROMs | Physical symptoms (no effect)<br>Patient-physician interaction (no effect) |
| Lewis et al [49,50] | 2010; 2011 | COPD | Self-report of symptoms, temperature, and oxygen levels twice daily | GP contacts (fewer), ED visits, hospital admissions, bed days, community COPD specialist visits (no effect)<br>QoL (no effect) |
| McCombie et al [51] | 2020 | IBD | Quarterly and as-needed self-report of symptoms and fecal calprotectin | Gastroenterology appointments (reduced)<br>QoL (non-inferior)<br>Physical symptoms (non-inferior) |

| | | | | |
|---|---|---|---|---|
| Ndosi et al [52] | 2016 | RA | Educational needs assessment before clinic visits | Physical symptoms (improvement) Self-efficacy (no effect) |
| Nguyen et al [53] | 2009 | COPD | Daily self-report of symptoms and exercise, text-message feedback, and telephone contact as needed | Clinical parameters (Decreased peak flow)<br>QoL (No effect)<br>Physical activity (decreased in 2/3 scales)<br>Self-efficacy (no effect) |
| Paré et al [54] | 2013 | COPD | Self-report of symptoms and use of medication on a tablet, automated self- management program with learning modules | Hospitalizations (fewer) ED visits (no effect) Respiratory therapists (no effect), Nurses' home visits (fewer)) |
| Park et al [55] | 2020 | COPD | Self-report of symptoms, use of medication and physical activity in a smart-phone app | Clinical parameters (no effect on exercise capacity)<br>Hospitalizations, ED visits (no effect) QoL (no effect)<br>Depression and anxiety (no effect) Self-efficacy (no effect)<br>Physical activity (improved) |
| Pers et al [56], Bernard et al [57] | 2021 | RA | Weekly self-report of symptoms and handgrip strength | Telephone contacts (increased) QoL (no effect on SF-12) Physical symptoms (no effect)<br>Mental health (no effect)<br>Satisfaction (no effect) |
| Piette et al [58] | 2001 | Diabetes | Automated phone calls with self-care education and assessment of blood glucose, self-care, and symptoms | Clinical parameters (positive effect on HbA1c in subgroups)<br>Podiatry and diabetes clinics visits (increased)<br>Physical symptoms (improved) Weight monitoring (no effect) Medication adherence (no effect)<br>Satisfaction (improved) |
| Piette et al [59,60] | 2000 | Diabetes | Biweekly automated phone calls with self-care education and assessment of blood glucose, self-care, and symptoms | Clinical parameters (reduced serum glucose, higher proportion with normal HbA1c)<br>Hospitalizations, outpatient visits (no effect)<br>Physical symptoms (improved) Depression (improved), anxiety (no effect)<br>Self-care, self-efficacy (improved), bad days (fewer)<br>Satisfaction (improved) |

| | | | | |
|---|---|---|---|---|
| Pinnock et al [61], Stoddart et al [62] | 2013 | COPD | Daily self-report of symptoms, use of treatment, and oxygen saturation | Mortality (no effect)<br>Bed days, time to admission, GP visits, nursing consultations (no effect) Clinical parameters (no effect on exacerbations)<br>QoL (No effect on EQ-5D)<br>Physical symptoms (no effect on SGRQ) Depression and anxiety (no effect)<br>Use of medication (no change) Satisfaction (no effect)<br>Cost (no difference in total cost and not cost-effective) |
| Pouwer et al [63] | 2001 | Diabetes | Psychological well-being questionnaire assessment and counselling in addition to usual clinic visits | |
| Pouwer et al [64] | 2011 | Diabetes | Diagnostic interview for mood and anxiety disorders. A diagnosis was followed by treatment advice to GP and diabetes specialist whereas absence of diagnosis was followed by self-management support. | Clinical parameters (no effect on HbA1c) Physical symptoms (improved) |
| Rassouli et al [65] | 2021 | COPD | Daily questions focused on recognizing acute exacerbation | Mortality (no effect)<br>ED visits, hospitalization (no effect) Clinical parameters (Increased detection of moderate exacerbations but not mild or severe)<br>Satisfaction (no effect)<br>Cost (no effect) |
| Schwarz et al [66] | 2008 | Heart disease | Daily self-report of weight, symptoms, medication, and health behavior | ED visits, days to readmission (no effect) QoL (no effect)<br>Depression (no effect) Caregiver mastery (no effect)<br>Cost (no effect) |
| Scollan- Koliopoulos et al [67] | 2012 | Diabetes | Brief needs-based assessment of emotional reactions to diabetes as supplement to clinic visit | Depression medication (no effect) |
| Sethares et al [68] | 2004 | Heart disease | Messages tailored to perceived benefits and barriers of self-care during and two times after hospitalization | QoL (no effect) |

| | | | | |
|---|---|---|---|---|
| Shara et al [69] | 2022 | Heart disease | Daily patient-report of self-care, medication, and symptoms through voice activated technology for 90 days | ED visits (increased) |
| Sink et al [70] | 2020 | COPD | Daily automated phone calls or text messages on breathing status with provider counselling in case of worsening | Hospitalization (fewer) |
| Slok et al [71] | 2016 | COPD | Pro interventions for COPD | Quality of care (improved) |
| Soran et al [72] | 2008 | Heart disease | Daily self-report of weight and symptoms in a telephone-connected monitor and personalized benchmarks | Mortality (no effect)<br>Hospitalization, ED visits, bed days (no effect)<br>Clinical parameters (no effect on invasive procedures)<br>Use of medication (no change) |
| Spaeder et al [73] | 2006 | Heart disease | Telephone-based automated daily questions on physiological parameters, symptoms, medication use and side effects during carvedilol titration | Mortality (no effect)<br>Use of medication (non-inferior of carvedilol)<br>Safety (non-inferior) |
| Subramanian et al [74], Keeffe et al [75] | 2004; 2005 | Heart disease | Patient-reported symptoms before general practice visits with medication suggestions | Hospitalization (increased) QoL (no effect)<br>Physical symptoms (no effect)<br>Satisfaction (increased) |
| van Dijk-de Vries et al [76] | 2015 | Diabetes | Detection and follow-up on mental health symptoms with self-management support in case of mental distress | Clinical parameters (no effect on HbA1c) QoL (no effect)<br>Participation, autonomy (no effect) |
| Vo et al [77] | 2019 | Diabetes | Patient-reported prioritization of concerns before clinic visit through a website | Visit interaction (improved) |
| Zakrisson et al [78] | 2020 | COPD | Patient-reported needs and priorities before clinic visit | General physical health (better QPP score)<br>Mental symptoms (no effect)<br>Personal attention (improved) |
| Östlund et al [79] | 2021 | IBD | Self-report of symptoms, disease activity, and fecal calprotectin upon suspected flare or remission | Healthcare interactions (no effect) Use of medication (increased) |

Abbreviations: PROM=patient-reported outcome measure, COPD=chronic obstructive pulmonary disease, IBD=inflammatory bowel disease, RA=rheumatoid arthritis, QoL=quality of life, GP=general practitioner, HRQoL=health related quality of life, ED=emergency department, EQ-5D=European Quality of life - 5 Dimensions, SF-36=The Short Form (36) Health Survey, HAQ=the Health Assessment Questionnaire, IBDQ-9=the 9-item inflammatory bowel disease questionnaire, HbA1c=Hemoglobin A1c, DAS28=the 28 joint disease activity score, PAID=the Problem Areas in Diabetes, SGRQ=St. George's Respiratory Questionnaire, QPP=the Quality from the Patient's Perspective Questionnaire

## References


1. Adams AS, Schmittdiel JA, Altschuler A, Bayliss EA, Neugebauer R, Ma L, et al. Automated symptom and treatment side effect monitoring for improved quality of life among adults with diabetic peripheral neuropathy in primary care: a pragmatic, cluster, randomized, controlled trial. Diabet Med. 2019 Jan;36(1):52-61. PMID: ClinicalTrials.gov/NCT02056431. doi: 10.1111/dme.13840.
2. Bentley CL, Mountain GA, Thompson J, Fitzsimmons DA, Lowrie K, Parker SG, et al. A pilot randomised controlled trial of a Telehealth intervention in patients with chronic obstructive pulmonary disease: challenges of clinician-led data collection. Trials. 2014 Aug 6;15:313. PMID: 25100550. doi: 10.1186/1745-6215-15-313.
3. Kargiannakis M, Fitzsimmons DA, Bentley CL, Mountain GA. Does Telehealth Monitoring Identify Exacerbations of Chronic Obstructive Pulmonary Disease and Reduce Hospitalisations? An Analysis of System Data. JMIR Med Inform. 2017 Mar 22;5(1):e8. PMID: 28330829. doi: 10.2196/medinform.6359.
4. Berinstein JA, Cohen-Mekelburg SA, Greenberg GM, Wray D, Berry SK, Saini SD, et al. A Care Coordination Intervention Improves Symptoms But Not Charges in High-Risk Patients With Inflammatory Bowel Disease. Clin Gastroenterol Hepatol. 2022 May;20(5):1029-38 e9. PMID: 34461298. doi: 10.1016/j.cgh.2021.08.034.
5. Berkhof FF, van den Berg JW, Uil SM, Kerstjens HA. Telemedicine, the effect of nurse-initiated telephone follow up, on health status and health-care utilization in COPD patients: a randomized trial. Respirology. 2015 Feb;20(2):279-85. PMID: 25400242. doi: 10.1111/resp.12437.
6. Bowles KH, Holland DE, Horowitz DA. A comparison of in-person home care, home care with telephone contact and home care with telemonitoring for disease management. J Telemed Telecare. 2009;15(7):344-50. PMID: 19815903. doi: 10.1258/jtt.2009.090118.
7. Boyne JJ, Vrijhoef HJ, Crijns HJ, De Weerd G, Kragten J, Gorgels AP. Tailored telemonitoring in patients with heart failure: results of a multicentre randomized controlled trial. Eur J Heart Fail. 2012 Jul;14(7):791-801. PMID: ClinicalTrials.gov/NCT00502255. doi: 10.1093/eurjhf/hfs058.
8. Boyne JJ, Vrijhoef HJ, Wit R, Gorgels AP. Telemonitoring in patients with heart failure, the TEHAF study: Study protocol of an ongoing prospective randomised trial. Int J Nurs Stud. 2011 Jan;48(1):94-9. PMID: 20615505. doi: 10.1016/j.ijnurstu.2010.05.017.
9. Boyne JJ, Vrijhoef HJ, Spreeuwenberg M, De Weerd G, Kragten J, Gorgels AP, et al. Effects of tailored telemonitoring on heart failure patients' knowledge, self-care, self-efficacy and adherence: a randomized controlled trial. Eur J Cardiovasc Nurs. 2014 Jun;13(3):243-52. PMID: 23630403. doi: 10.1177/1474515113487464.
10. Gingele AJ, Ramaekers B, Brunner-La Rocca HP, De Weerd G, Kragten J, van Empel V, et al. Effects of tailored telemonitoring on functional status and health-related quality of life in patients with heart failure. Neth Heart J. 2019 Nov;27(11):565-74. PMID: 31414308. doi: 10.1007/s12471-019-01323-x.
11. Ramaekers BL, Janssen-Boyne JJ, Gorgels AP, Vrijhoef HJ. Adherence among telemonitored patients with heart failure to pharmacological and nonpharmacological recommendations. Telemed J E Health. 2009 Jul-Aug;15(6):517-24. PMID: 19566401. doi: 10.1089/tmj.2009.0160.
12. Chaudhry SI, Mattera JA, Curtis JP, Spertus JA, Herrin J, Lin Z, et al. Telemonitoring in patients with heart failure. N Engl J Med. 2010 Dec 9;363(24):2301-9. PMID: ClinicalTrials.gov/NCT00303212. doi: 10.1056/NEJMoa1010029.
13. Jayaram NM, Khariton Y, Krumholz HM, Chaudhry SI, Mattera J, Tang F, et al. Impact of Telemonitoring on Health Status. Circ Cardiovasc Qual Outcomes. 2017 Dec;10(12). PMID: ClinicalTrials.gov/NCT00303212. doi: 10.1161/CIRCOUTCOMES.117.004148.

14. Cordova FC, Ciccolella D, Grabianowski C, Gaughan J, Brennan K, Goldstein F, et al. A Telemedicine-Based Intervention Reduces the Frequency and Severity of COPD Exacerbation Symptoms: A Randomized, Controlled Trial. Telemed J E Health. 2016 Feb;22(2):114-22. PMID: 26259074. doi: 10.1089/tmj.2015.0035.
15. Cross RK, Cheevers N, Rustgi A, Langenberg P, Finkelstein J. Randomized, controlled trial of home telemanagement in patients with ulcerative colitis (UC HAT). Inflamm Bowel Dis. 2012 Jun;18(6):1018-25. PMID: 21688350. doi: 10.1002/ibd.21795.
16. Cross RK, Langenberg P, Regueiro M, Schwartz DA, Tracy JK, Collins JF, et al. A Randomized Controlled Trial of TELEmedicine for Patients with Inflammatory Bowel Disease (TELE-IBD). Am J Gastroenterol. 2019 Mar;114(3):472-82. PMID: 30410041. doi: 10.1038/s41395-018-0272-8.
17. Bilgrami Z, Abutaleb A, Chudy-Onwugaje K, Langenberg P, Regueiro M, Schwartz DA, et al. Effect of TELEmedicine for Inflammatory Bowel Disease on Patient Activation and Self-Efficacy. Dig Dis Sci. 2020 Jan;65(1):96-103. PMID: 30604373. doi: 10.1007/s10620-018-5433-5.
18. Bilgrami Z, Abutaleb A, Chudy-Onwugaje K, Langenberg P, Regueiro M, Schwartz DA, et al. Correction to: Effect of TELEmedicine for Inflammatory Bowel Disease on Patient Activation and Self-Efficacy (Digestive Diseases and Sciences, (2020), 65, 1, (96-103), 10.1007/s10620-018-5433-5). Digestive Diseases and Sciences. 2020 Feb;65(2):668. PMID: 31792670. doi: http://dx.doi.org/10.1007/s10620-019-05953-4.
19. Quinn CC, Chard S, Roth EG, Eckert JK, Russman KM, Cross RK. The Telemedicine for Patients With Inflammatory Bowel Disease (TELE-IBD) Clinical Trial: Qualitative Assessment of Participants' Perceptions. J Med Internet Res. 2019 Jun 3;21(6):e14165. PMID: 31162128. doi: 10.2196/14165.
20. Schliep M, Chudy-Onwugaje K, Abutaleb A, Langenberg P, Regueiro M, Schwartz DA, et al. TELEmedicine for Patients With Inflammatory Bowel Disease (TELE-IBD) Does Not Improve Depressive Symptoms or General Quality of Life Compared With Standard Care at Tertiary Referral Centers. Crohns Colitis 360. 2020 Jan;2(1):otaa002. PMID: 32201859. doi: 10.1093/crocol/otaa002.
21. Dang S, Karanam C, Gómez-Marín O. Outcomes of a Mobile Phone Intervention for Heart Failure in a Minority County Hospital Population. Telemed J E Health. 2017 Jun;23(6):473-84. PMID: 28051357. doi: 10.1089/tmj.2016.0211.
22. Dang S, Karanam C, Gómez-Orozco C, Gómez-Marín O. Mobile Phone Intervention for Heart Failure in a Minority Urban County Hospital Population: Usability and Patient Perspectives. Telemed J E Health. 2017 Jul;23(7):544-54. PMID: 28051761. doi: 10.1089/tmj.2016.0224.
23. Dansky K, Vasey J. Managing heart failure patients after formal homecare. Telemed J E Health. 2009 Dec;15(10):983-91. PMID: 19929234. doi: 10.1089/tmj.2009.0064.
24. de Jong MJ, Boonen A, van der Meulen-de Jong AE, Romberg-Camps MJ, van Bodegraven AA, Mahmmod N, et al. Cost-effectiveness of Telemedicine-directed Specialized vs Standard Care for Patients With Inflammatory Bowel Diseases in a Randomized Trial. Clin Gastroenterol Hepatol. 2020 Jul;18(8):1744-52. PMID: ClinicalTrials.gov/NCT02173002. doi: 10.1016/j.cgh.2020.04.038.
25. de Jong MJ, van der Meulen-de Jong AE, Romberg-Camps MJ, Becx MC, Maljaars JP, Cilissen M, et al. Telemedicine for management of inflammatory bowel disease (myIBDcoach): a pragmatic, multicentre, randomised controlled trial. Lancet. 2017 Sep 2;390(10098):959-68. PMID: ClinicalTrials.gov/NCT02173002. doi: 10.1016/S0140-6736(17)31327-2.
26. de Thurah A, Stengaard-Pedersen K, Axelsen M, Fredberg U, Schougaard LMV, Hjollund NHI, et al. Tele-Health Followup Strategy for Tight Control of Disease Activity in Rheumatoid Arthritis: Results of a Randomized Controlled Trial. Arthritis Care Res (Hoboken). 2018 Mar;70(3):353-60. PMID: ClinicalTrials.gov/NCT02155894. doi: 10.1002/acr.23280.

27. Skovsgaard CV, Kruse M, Hjollund NHI, Maribo T, de Thurah A. Cost-effectiveness of a telehealth intervention in rheumatoid arthritis: economic evaluation of the Telehealth in RA (TeRA) randomized controlled trial. Scandinavian Journal of Rheumatology. 2023 2023/03/04;52(2):118-28. doi: 10.1080/03009742.2021.2008604.
28. Del Hoyo J, Nos P, Faubel R, Muñoz D, Domínguez D, Bastida G, et al. A Web-Based Telemanagement System for Improving Disease Activity and Quality of Life in Patients With Complex Inflammatory Bowel Disease: Pilot Randomized Controlled Trial. J Med Internet Res. 2018 Nov 27;20(11):e11602. PMID: ClinicalTrials.gov/NCT02943538. doi: 10.2196/11602.
29. Del Hoyo J, Nos P, Bastida G, Faubel R, Muñoz D, Garrido-Marín A, et al. Telemonitoring of Crohn's Disease and Ulcerative Colitis (TECCU): Cost-Effectiveness Analysis. J Med Internet Res. 2019 Sep 13;21(9):e15505. PMID: ClinicalTrials.gov/NCT02943538. doi: 10.2196/15505.
30. Elkjaer M, Shuhaibar M, Burisch J, Bailey Y, Scherfig H, Laugesen B, et al. E-health empowers patients with ulcerative colitis: a randomised controlled trial of the web-guided 'Constant-care' approach. Gut. 2010 Dec;59(12):1652-61. PMID: 21071584. doi: 10.1136/gut.2010.220160.
31. Farmer A, Williams V, Velardo C, Shah SA, Yu LM, Rutter H, et al. Self-Management Support Using a Digital Health System Compared With Usual Care for Chronic Obstructive Pulmonary Disease: Randomized Controlled Trial. J Med Internet Res. 2017 May 3;19(5):e144. PMID: ISRCTN/ISRCTN40367841. doi: 10.2196/jmir.7116.
32. Velardo C, Shah SA, Gibson O, Clifford G, Heneghan C, Rutter H, et al. Digital health system for personalised COPD long-term management. BMC Med Inform Decis Mak. 2017 Feb 20;17(1):19. PMID: ISRCTN/ISRCTN40367841. doi: 10.1186/s12911-017-0414-8.
33. Whelan ME, Velardo C, Rutter H, Tarassenko L, Farmer AJ. Mood Monitoring Over One Year for People With Chronic Obstructive Pulmonary Disease Using a Mobile Health System: Retrospective Analysis of a Randomized Controlled Trial. JMIR Mhealth Uhealth. 2019 Nov 22;7(11):e14946. PMID: 31755872. doi: 10.2196/14946.
34. Frasure-Smith N, Prince RH. The Ischemic Heart Disease Life Stress Monitoring Program: 18-Month mortality results. Canadian Journal of Public Health. 1986 May-Jun;77(SUPPL. 1):46-50. PMID: 3527398.
35. Frasure-Smith N, Lespérance F, Prince RH, Verrier P, Garber RA, Juneau M, et al. Randomised trial of home-based psychosocial nursing intervention for patients recovering from myocardial infarction. Lancet. 1997 Aug 16;350(9076):473-9. PMID: 9274583. doi: 10.1016/S0140-6736(97)02142-9.
36. GESICA Investigators. Randomised trial of telephone intervention in chronic heart failure: DIAL trial. BMJ. 2005 Aug 20;331(7514):425. PMID: 16061499. doi: 10.1136/bmj.38516.398067.E0.
37. Goldberg LR, Piette JD, Walsh MN, Frank TA, Jaski BE, Smith AL, et al. Randomized trial of a daily electronic home monitoring system in patients with advanced heart failure: the Weight Monitoring in Heart Failure (WHARF) trial. Am Heart J. 2003 Oct;146(4):705-12. PMID: 14564327. doi: 10.1016/S0002-8703(03)00393-4.
38. Hernar I, Graue M, Richards DA, Strandberg RB, Nilsen RM, Rekdal M, et al. Use of patient-reported outcome measures (PROMs) in clinical diabetes consultations: the DiaPROM randomised controlled pilot trial. BMJ Open. 2021 Apr 14;11(4):e042353. PMID: 33853796. doi: 10.1136/bmjopen-2020-042353.
39. Hueppe A, Langbrandtner J, Raspe H. Inviting patients with inflammatory bowel disease to active involvement in their own care: a randomized controlled trial. Inflamm Bowel Dis. 2014 Jun;20(6):1057-69. PMID: 24788217. doi: 10.1097/MIB.0000000000000044.
40. Huffstutter J, Craig WD, Schimizzi G, Harshbarger J, Lisse J, Kasle S, et al. A multicenter, randomized, open study to evaluate the impact of an electronic data capture system on the care of patients with rheumatoid arthritis. Curr Med Res Opin. 2007 Aug;23(8):1967-79. PMID: 17626700. doi: 10.1185/030079907X210624.

41. Johnson AE, Routh S, Taylor CN, Leopold M, Beatty K, McNamara DM, et al. Developing and Implementing an mHealth Heart Failure Self-care Program to Reduce Readmissions: Randomized Controlled Trial. JMIR Cardio. 2022 2022/3/21;6(1):e33286. doi: 10.2196/33286.
42. Kronish IM, Moise N, Cheung YK, Clarke GN, Dolor RJ, Duer-Hefele J, et al. Effect of Depression Screening after Acute Coronary Syndromes on Quality of Life: The CODIACS-QoL Randomized Clinical Trial. JAMA Internal Medicine. 2020 Jan 1;180(1):45-53. PMID: 31633746. doi: http://dx.doi.org/10.1001/jamainternmed.2019.4518.
43. Ladapo JA, Davidson KW, Moise N, Chen A, Clarke GN, Dolor RJ, et al. Economic outcomes of depression screening after acute coronary syndromes: The CODIACS-QoL randomized clinical trial. General Hospital Psychiatry. 2021 2021/07/01/;71:47-54. doi: https://doi.org/10.1016/j.genhosppsych.2021.04.001.
44. Krum H, Forbes A, Yallop J, Driscoll A, Croucher J, Chan B, et al. Telephone support to rural and remote patients with heart failure: the Chronic Heart Failure Assessment by Telephone (CHAT) study. Cardiovasc Ther. 2013 Aug;31(4):230-7. PMID: 23061492. doi: 10.1111/1755-5922.12009.
45. Kuusalo L, Sokka-Isler T, Kautiainen H, Ekman P, Kauppi MJ, Pirilä L, et al. Automated Text Message-Enhanced Monitoring Versus Routine Monitoring in Early Rheumatoid Arthritis: A Randomized Trial. Arthritis Care Res (Hoboken). 2020 Mar;72(3):319-25. PMID: ClinicalTrials.gov/NCT02424877. doi: 10.1002/acr.23846.
46. Laurberg T, Schougaard LMV, Hjollund NHI, Lomborg KE, Hansen TK, Jensen AL. Randomized controlled study to evaluate the impact of flexible patient-controlled visits in people with type 1 diabetes: The DiabetesFlex Trial. Diabetic Medicine. 2022;39(5):e14791. doi: https://doi.org/10.1111/dme.14791.
47. Lee Y, Lu F, Colls J, Murray M, Suh D, Song J, et al. Effect of a mobile app to monitor patient reported outcomes in rheumatoid arthritis: A randomized controlled trial. Arthritis and Rheumatology. 2019;71(Supplement 10):1430-1. doi: http://dx.doi.org/10.1002/art.41108.
48. Colls J, Lee Y, Xu C, Corrigan C, Lu F, Marquez-Grap G, Murray M, Suh DH, Solomon DH. Patient adherence with a smartphone app for patient-reported outcomes in rheumatoid arthritis. Rheumatology (Oxford) 2021 Jan 05; 60(1):108-112. PMID: 32572490. doi: 10.1093/rheumatology/keaa202.
49. Lewis KE, Annandale JA, Warm DL, Hurlin C, Lewis MJ, Lewis L. Home telemonitoring and quality of life in stable, optimised chronic obstructive pulmonary disease. J Telemed Telecare. 2010;16(5):253-9. PMID: 20483881. doi: 10.1258/jtt.2009.090907.
50. Lewis KE, Annandale JA, Warm DL, Rees SE, Hurlin C, Blyth H, et al. Does home telemonitoring after pulmonary rehabilitation reduce healthcare use in optimized COPD a pilot randomized trial. COPD: Journal of Chronic Obstructive Pulmonary Disease. 2011 Feb;7(1):44-50. PMID: 20214462. doi: http://dx.doi.org/10.3109/15412550903499555.
51. McCombie A, Walmsley R, Barclay M, Ho C, Langlotz T, Regenbrecht H, et al. A Noninferiority Randomized Clinical Trial of the Use of the Smartphone-Based Health Applications IBDsmart and IBDoc in the Care of Inflammatory Bowel Disease Patients. Inflamm Bowel Dis. 2020 Jun 18;26(7):1098-109. PMID: 31644793. doi: 10.1093/ibd/izz252.
52. Ndosi M, Johnson D, Young T, Hardware B, Hill J, Hale C, et al. Effects of needs-based patient education on self-efficacy and health outcomes in people with rheumatoid arthritis: a multicentre, single blind, randomised controlled trial. Ann Rheum Dis. 2016 Jun;75(6):1126-32. PMID: 26162769. doi: 10.1136/annrheumdis-2014-207171.
53. Nguyen HQ, Gill DP, Wolpin S, Steele BG, Benditt JO. Pilot study of a cell phone-based exercise persistence intervention post-rehabilitation for COPD. Int J Chron Obstruct Pulmon Dis. 2009;4:301-13. PMID: 19750190. doi: 10.2147/copd.s6643.
54. Paré G, Poba-Nzaou P, Sicotte C, Beaupre A, Lefrancois E, Nault D, et al. Comparing the costs of home telemonitoring and usual care of chronic obstructive pulmonary disease patients: A randomized controlled trial. European Research in Telemedicine. 2013;2(2):35-47. doi: http://dx.doi.org/10.1016/j.eurtel.2013.05.001.

55. Park SK, Bang CH, Lee SH. Evaluating the effect of a smartphone app-based self-management program for people with COPD: A randomized controlled trial. Appl Nurs Res. 2020 Apr;52:151231. PMID: 31955942. doi: 10.1016/j.apnr.2020.151231.
56. Pers YM, Valsecchi V, Mura T, Aouinti S, Filippi N, Marouen S, et al. A randomized prospective open-label controlled trial comparing the performance of a connected monitoring interface versus physical routine monitoring in patients with rheumatoid arthritis. Rheumatology (Oxford). 2021 Apr 6;60(4):1659-68. PMID: 33020846. doi: 10.1093/rheumatology/keaa462.
57. Bernard L, Valsecchi V, Mura T, Aouinti S, Padern G, Ferreira R, et al. Management of patients with rheumatoid arthritis by telemedicine: connected monitoring. A randomized controlled trial. Joint Bone Spine. 2022 2022/10/01/;89(5):105368. doi: https://doi.org/10.1016/j.jbspin.2022.105368.
58. Piette JD, Weinberger M, Kraemer FB, McPhee SJ. Impact of automated calls with nurse follow-up on diabetes treatment outcomes in a Department of Veterans Affairs Health Care System: a randomized controlled trial. Diabetes Care. 2001 Feb;24(2):202-8. PMID: 11213866. doi: 10.2337/diacare.24.2.202.
59. Piette JD, Weinberger M, McPhee SJ. The effect of automated calls with telephone nurse follow-up on patient-centered outcomes of diabetes care: a randomized, controlled trial. Med Care. 2000 Feb;38(2):218-30. PMID: 10659695. doi: 10.1097/00005650-200002000-00011.
60. Piette JD, Weinberger M, McPhee SJ, Mah CA, Kraemer FB, Crapo LM. Do automated calls with nurse follow-up improve self-care and glycemic control among vulnerable patients with diabetes? Am J Med. 2000;108(1):20-7. doi: 10.1016/s0002-9343(99)00298-3.
61. Pinnock H, Hanley J, McCloughan L, Todd A, Krishan A, Lewis S, et al. Effectiveness of telemonitoring integrated into existing clinical services on hospital admission for exacerbation of chronic obstructive pulmonary disease: researcher blind, multicentre, randomised controlled trial. BMJ. 2013 Oct 17;347:f6070. PMID: ISRCTN/ISRCTN96634935. doi: 10.1136/bmj.f6070.
62. Stoddart A, van der Pol M, Pinnock H, Hanley J, McCloughan L, Todd A, et al. Telemonitoring for chronic obstructive pulmonary disease: a cost and cost-utility analysis of a randomised controlled trial. J Telemed Telecare. 2015 Mar;21(2):108-18. PMID: 25586810. doi: 10.1177/1357633X14566574.
63. Pouwer F, Snoek FJ, van der Ploeg HM, Adèr HJ, Heine RJ. Monitoring of psychological well-being in outpatients with diabetes: effects on mood, HbA(1c), and the patient's evaluation of the quality of diabetes care: a randomized controlled trial. Diabetes Care. 2001 Nov;24(11):1929-35. PMID: 11679459. doi: 10.2337/diacare.24.11.1929.
64. Pouwer F, Tack CJ, Geelhoed-Duijvestijn PH, Bazelmans E, Beekman AT, Heine RJ, et al. Limited effect of screening for depression with written feedback in outpatients with diabetes mellitus: a randomised controlled trial. Diabetologia. 2011 Apr;54(4):741-8. PMID: 21221528. doi: 10.1007/s00125-010-2033-0.
65. Rassouli F, Germann A, Baty F, Kohler M, Stolz D, Thurnheer R, et al. Telehealth mitigates COPD disease progression compared to standard of care: a randomized controlled crossover trial. J Intern Med. 2021 Mar;289(3):404-10. PMID: 33428219. doi: 10.1111/joim.13230.
66. Schwarz KA, Mion LC, Hudock D, Litman G. Telemonitoring of heart failure patients and their caregivers: a pilot randomized controlled trial. Prog Cardiovasc Nurs. 2008 Winter;23(1):18-26. PMID: 18326990. doi: 10.1111/j.1751-7117.2008.06611.x.
67. Scollan-Koliopoulos M, Herrera I, Romano K, Gregory C, Rapp K, Bleich D. Healthcare Technician Delivered Screening of Adults with Diabetes to Improve Primary Care Provider Recognition of Depression. Journal of Family Medicine and Primary Care. 2012 July 1, 2012;1(2):97-102. PMID: 24479015. doi: 10.4103/2249-4863.104955.
68. Sethares KA, Elliott K. The effect of a tailored message intervention on heart failure readmission rates, quality of life, and benefit and barrier beliefs in persons with heart failure. Heart Lung. 2004 Jul-Aug;33(4):249-60. PMID: 15252415. doi: 10.1016/j.hrtlng.2004.03.005.
69. Shara N, Bjarnadottir MV, Falah N, Chou J, Alqutri HS, Asch FM, et al. Voice activated remote monitoring technology for heart failure patients: Study design, feasibility and observations from a pilot randomized control trial. PLOS ONE. 2022;17(5):e0267794. doi: 10.1371/journal.pone.0267794.

70. Sink E, Patel K, Groenendyk J, Peters R, Som A, Kim E, et al. Effectiveness of a novel, automated telephone intervention on time to hospitalisation in patients with COPD: A randomised controlled trial. J Telemed Telecare. 2020 Apr;26(3):132-9. PMID: 30269640. doi: 10.1177/1357633X18800211.
71. Slok AH, Kotz D, van Breukelen G, Chavannes NH, Rutten-van Mölken MP, Kerstjens HA, et al. Effectiveness of the Assessment of Burden of COPD (ABC) tool on health-related quality of life in patients with COPD: a cluster randomised controlled trial in primary and hospital care. BMJ Open. 2016 Jul 11;6(7):e011519. PMID: 27401361. doi: 10.1136/bmjopen-2016-011519.
72. Soran OZ, Pina IL, Lamas GA, Kelsey SF, Selzer F, Pilotte J, et al. A Randomized Clinical Trial of the Clinical Effects of Enhanced Heart Failure Monitoring Using a Computer-Based Telephonic Monitoring System in Older Minorities and Women. Journal of Cardiac Failure. 2008 Nov;14(9):711-7. PMID: 18995174. doi: http://dx.doi.org/10.1016/j.cardfail.2008.06.448.
73. Spaeder J, Najjar SS, Gerstenblith G, Hefter G, Kern L, Palmer JG, et al. Rapid titration of carvedilol in patients with congestive heart failure: a randomized trial of automated telemedicine versus frequent outpatient clinic visits. Am Heart J. 2006 Apr;151(4):844.e1-10. PMID: 16569544. doi: 10.1016/j.ahj.2005.06.044.
74. Subramanian U, Fihn SD, Weinberger M, Plue L, Smith FE, Udris EM, et al. A controlled trial of including symptom data in computer-based care suggestions for managing patients with chronic heart failure. Am J Med. 2004 Mar 15;116(6):375-84. PMID: 15006586. doi: 10.1016/j.amjmed.2003.11.021.
75. Keeffe B, Subramanian U, Tierney WM, Udris E, Willems J, McDonell M, et al. Provider response to computer-based care suggestions for chronic heart failure. Med Care. 2005 May;43(5):461-5. PMID: 15838410. doi: 10.1097/01.mlr.0000160378.53326.f3.
76. van Dijk-de Vries A, van Bokhoven MA, Winkens B, Terluin B, Knottnerus JA, van der Weijden T, et al. Lessons learnt from a cluster-randomised trial evaluating the effectiveness of Self-Management Support (SMS) delivered by practice nurses in routine diabetes care. BMJ Open. 2015 Jun 25;5(6):e007014. PMID: NTR/NTR2764. doi: 10.1136/bmjopen-2014-007014.
77. Vo MT, Uratsu CS, Estacio KR, Altschuler A, Kim E, Alexeeff SE, et al. Prompting Patients with Poorly Controlled Diabetes to Identify Visit Priorities Before Primary Care Visits: a Pragmatic Cluster Randomized Trial. J Gen Intern Med. 2019 Jun;34(6):831-8. PMID: ClinicalTrials.gov/NCT02375932. doi: 10.1007/s11606-018-4756-4.
78. Zakrisson AB, Arne M, Lisspers K, Lundh L, Sandelowsky H, Stallberg B, et al. Improved quality of care by using the PRISMS form to support self-management in patients with COPD: A Randomised Controlled Trial. J Clin Nurs. 2020 Jul;29(13-14):2410-9. PMID: 32220091. doi: 10.1111/jocn.15253.
79. östlund I, Werner M, Karling P. Self-monitoring with home based fecal calprotectin is associated with increased medical treatment. A randomized controlled trial on patients with inflammatory bowel disease. Scand J Gastroenterol. 2021 Jan;56(1):38-45. PMID: 33284639. doi: 10.1080/00365521.2020.1854342.
